# Supplementary material for: An orthogonal transcription mutation system generating all transition mutations for accelerated protein evolution in vivo
Source: Nat Commun. 2025 Jul 1;16:6041. doi: 10.1038/s41467-025-61354-4 (PMC12218169; doi:10.1038/s41467-025-61354-4)
Supplement: Supplementary file 1 — Supplementary Information [file 41467_2025_61354_MOESM1_ESM.pdf]

Title:

An Orthogonal Transcription Mutation System Generating All Transition Mutations for Accelerated Protein Evolution *in vivo*

Supplementary Table 1. Strains used in this study.

Strain	Description	Reference
Escherichia coli S17-1	A plasmid donor for conjugation, carries *tra* genes from RP4 plasmid in the genome	[1]
E. coli MG1655	K-12 (F- *lambda*- rph-1)	[2]
E. coli MG1655-*mreBCD*-P_{MmP1}	*E. coli* MG1655 derivative with a reverse P_{MmP1} promoter placed downstream of the *mreBCD* gene cluster in the genome	This study
E. coli MG1655-*ftsQAZ*-P_{MmP1}	*E. coli* MG1655 derivative with a reverse P_{MmP1} promoter placed downstream of the *ftsQAZ* gene cluster in the genome	This study
Halomonas bluephagenesis TD01	*Halomonas bluephagenesis* TD wildtype, isolated from Aydingol Lake in China	[3]
H. bluephagenesis TD01Δ*IS30*	*H. bluephagenesis* TD01 derivative with six IS30 family transposases deleted	This study
H. bluephagenesis TD01-G4-*sacB*-P_{MmP1}	*H. bluephagenesis* TD01 integrated "$P_{Porin42}$-*sacB*-P_{MmP1} (reverse)" on G4 site	This study
H. bluephagenesis TD01-G7-*sacB*-P_{MmP1}	*H. bluephagenesis* TD01 integrated "$P_{Porin42}$-*sacB*-P_{MmP1} (reverse)" on G7 site	This study
H. bluephagenesis TD01-G7-P_{MmP1}-*sacB*-P_{MmP1}	*H. bluephagenesis* TD01 integrated "P_{MmP1}-$P_{Porin42}$-*sacB*-P_{MmP1} (reverse)" on G7 site	This study
H. bluephagenesis TD01-G7-P_{K1F}-*sacB*-P_{K1F}	*H. bluephagenesis* TD01 integrated "P_{K1F}-$P_{Porin42}$-*sacB*-P_{K1F} (reverse)" on G7 site	This study
H. bluephagenesis TD01-G7-P_{VP4}-*sacB*-P_{VP4}	*H. bluephagenesis* TD01 integrated "P_{VP4}-$P_{Porin42}$-*sacB*-P_{VP4} (reverse)" on G7 site	This study
H. bluephagenesis TD01-*mreBCD*-P_{MmP1}	*H. bluephagenesis* TD01 derivative with a reverse P_{MmP1} promoter downstream of *mreBCD* gene cluster in the genome	This study
H. bluephagenesis TD01-*ftsQAZ*-P_{MmP1}	*H. bluephagenesis* TD01 derivative with a reverse P_{MmP1} promoter placed downstream of the *ftsQAZ* gene cluster in the genome	This study
H. bluephagenesis TD01-*mreBCD&ftsQAZ*-P_{MmP1}	*H. bluephagenesis* TD01 derivative with two reverse P_{MmP1} promoters placed downstream of *mreBCD* and *ftsQAZ* gene clusters in the genome, respectively	This study

Supplementary Table 2. Plasmids used in this study.

Plasmid	Description	Reference
pSEVA241	A broad-host expression vector, pRO1600/ColE1 replication origin, high copy-number, oriT, Kan^R, Spe^R.	[4]
p241	pSEVA241 derivate, empty vector	This study
pMT0-MmP1	pSEVA241 derivate, expressing MmP1 RNAP controlled by P_{Tac} promoter	This study
pMT1-MmP1	pSEVA241 derivate, expressing PmCDA1-MmP1-RNAP controlled by P_{Tac} promoter	This study
pMT2-MmP1	pSEVA241 derivate, expressing PmCDA1-UGI-MmP1-RNAP controlled by P_{Tac} promoter	This study
pMT2-MmP1-S	pSEVA241 derivate, expressing PmCDA1-MmP1-RNAP and UGI (strong RBS) controlled by P_{Tac} promoter	This study
pMT2-MmP1-M	pSEVA241 derivate, expressing PmCDA1-MmP1-RNAP and UGI (medium RBS) controlled by P_{Tac}c promoter	This study
pMT2-MmP1-W	pSEVA241 derivate, expressing PmCDA1-MmP1-RNAP and UGI (weak RBS) controlled by P_{Tac} promoter	This study
pMT2.1-MmP1	pSEVA241 derivate, expressing evoPmCDA1-UGI-MmP1-RNAP controlled by P_{Tac} promoter	This study
pMT3-MmP1	pSEVA241 derivate, expressing TadA8e-MmP1-RNAP controlled by P_{Tac} promoter	This study
pMT3.1-MmP1	pSEVA241 derivate, expressing TadA7.10-MmP1-RNAP controlled by P_{Tac} promoter	This study
pMT3.2-MmP1	pSEVA241 derivate, expressing CABE T3.1-MmP1-RNAP controlled by P_{Tac} promoter	This study
pMT3.3-MmP1	pSEVA241 derivate, expressing CABE T3.155-MmP1-RNAP controlled by P_{Tac} promoter	This study
pMT3.4-MmP1	pSEVA241 derivate, expressing TadA9-MmP1-RNAP controlled by P_{Tac} promoter	This study
pMT3.5-MmP1	pSEVA241 derivate, expressing TadDE-MmP1-RNAP controlled by P_{Tac} promoter	This study
pMT0-K1F	pSEVA241 derivate, expressing K1F RNAP controlled by P_{Tac} promoter	This study
pMT2-K1F	pSEVA241 derivate, expressing PmCDA1-UGI-K1F-RNAP controlled by P_{Tac} promoter	This study
pMT3-K1F	pSEVA241 derivate, expressing TadA8e-K1F-RNAP controlled by P_{Tac} promoter	This study
pMT0-VP4	pSEVA241 derivate, expressing VP4 RNAP controlled by P_{Tac} promoter	This study
pMT2-VP4	pSEVA241 derivate, expressing PmCDA1-UGI-VP4-RNAP controlled by P_{Tac} promoter	This study
pMT3-VP4	pSEVA241 derivate, expressing TadA8e-VP4-RNAP controlled by P_{Tac} promoter	This study
pMT23d-MmP1	pSEVA241 derivate, expressing PmCDA1-UGI-TadA8e-MmP1-RNAP controlled by P_{Tac} promoter	This study

pMT32d-MmP1	pSEVA241 derivate, expressing TadA8e-PmCDA1-UGI-MmP1-RNAP controlled by P_{Tac} promoter	This study
pMT23opt-MmP1	pSEVA241 derivate, expressing PmCDA1-UGI-MmP1-RNAP and TadA8e-MmP1-RNAP (opt) controlled by P_{Tac} promoter	This study
pMT23d-K1F	pSEVA241 derivate, expressing PmCDA1-UGI-TadA8e-K1F-RNAP controlled by P_{Tac} promoter	This study
pMT32d-K1F	pSEVA241 derivate, expressing TadA8e-PmCDA1-UGI-K1F-RNAP controlled by P_{Tac} promoter	This study
pMT23opt-K1F	pSEVA241 derivate, expressing PmCDA1-UGI-K1F-RNAP and TadA8e-K1F-RNAP (opt) controlled by P_{Tac} promoter	This study
pMT23d-VP4	pSEVA241 derivate, expressing PmCDA1-UGI-TadA8e-VP4-RNAP controlled by P_{Tac} promoter	This study
pMT32d-VP4	pSEVA241 derivate, expressing TadA8e-PmCDA1-UGI-VP4-RNAP controlled by P_{Tac} promoter	This study
pMT23opt-VP4	pSEVA241 derivate, expressing PmCDA1-UGI-VP4-RNAP and TadA8e-VP4-RNAP (opt) controlled by P_{Tac} promoter	This study
pSEVA321	An expression vector in *H. bluephagenesis* and *E. col*, RK2 replication origin, low copy-number, oriT, Cm^R	[4]
pMT11-MmP1	pSEVA241 derivate, expressing sfGFP controlled by P_{MmP1} promoter	This study
pMT15-MmP1	pSEVA321 derivate, expressing "P_{MmP1}-*ermC* wild-type"	This study
pMT37-MmP1	pSEVA321 derivate, expressing "$P_{Porin42}$-*sacB*-P_{MmP1} (reverse)"	This study
pMT38-MmP1	pSEVA321 derivate, expressing "P_{MmP1}-$P_{Porin42}$-*sacB*-P_{MmP1} (reverse)"	This study
pMT38-K1F	pSEVA321 derivate, expressing "P_{K1F}-$P_{Porin42}$-*sacB*-P_{K1F} (reverse)"	This study
pMT38-VP4	pSEVA321 derivate, expressing "P_{VP4}-$P_{Porin42}$-*sacB*-P_{VP4} (reverse)"	This study
pMT43-MmP1	pSEVA321 derivate, expressing "P_{MmP1}-*ermC* Y104H"	This study
pMT44-MmP1	pSEVA321 derivate, expressing "P_{MmP1}-*ermC* Y104F"	This study
pMT45-MmP1	pSEVA321 derivate, expressing "P_{MmP1}-*ermC* Y104L"	This study
pMT46-MmP1	pSEVA321 derivate, expressing "P_{MmP1}-*ermC* Y104S"	This study
pMT49-MmP1	pSEVA321 derivate, expressing "P_{MmP1}-$P_{Porin42}$-*ermC* wild-type"	This study
pMT58-MmP1	pSEVA321 derivate, expressing "P_{MmP1}-$P_{Porin42}$-*ermC* Q10*"	This study
pMT58-K1F	pSEVA321 derivate, expressing "P_{K1F}-$P_{Porin42}$-*ermC* Q10*"	This study
pMT58-VP4	pSEVA321 derivate, expressing "P_{VP4}-$P_{Porin42}$-*ermC* Q10*"	This study
pMT59-MmP1	pSEVA321 derivate, expressing "P_{MmP1}-$P_{Porin42}$-*ermC* Q79*"	This study
pMT60-MmP1	pSEVA321 derivate, expressing "P_{MmP1}-$P_{Porin42}$-*ermC* Q87*"	This study
pMT61-MmP1	pSEVA321 derivate, expressing "P_{MmP1}-$P_{Porin42}$-*ermC* R21*"	This study
pMT62-MmP1	pSEVA321 derivate, expressing "P_{MmP1}-$P_{Porin42}$-*ermC* R51*"	This study
pMT63-MmP1	pSEVA321 derivate, expressing "P_{MmP1}-$P_{Porin42}$-*ermC* R134*"	This study

pMT64-MmP1	pSEVA321 derivate, expressing "P_{MmP1}-$P_{Porin42}$-*ermC* Y104S"	This study
pMT64-K1F	pSEVA321 derivate, expressing "P_{K1F}-$P_{Porin42}$-*ermC* Y104S"	This study
pMT64-VP4	pSEVA321 derivate, expressing "P_{VP4}-$P_{Porin42}$-*ermC* Y104S"	This study
pMT85-MmP1	pSEVA321 derivate, expressing "$P_{Porin141}$-P_{MmP1}-*mCheery-sfGFP-TagBFP*-P_{MmP1} (reverse)"	This study
pMT86-MmP1	pSEVA321 derivate, expressing "$P_{Porin141}$-P_{MmP1}- *amajLime-fwYellow-spisPink*-P_{MmP1} (reverse)"	This study
pHbPBC	A stable recombinant endogenous vector in *H. bluephagenesis*, pHbPBC/ColE1 replicon, low copy-number, oriT, hbpB/hbpC toxin/antitoxin system, Cm^R	[5]
pMT91-MmP1	pHbPBC derivate, expressing "P_{MmP1}-P_{rpoD}-*rpoD*-P_{MmP1} (reverse)"	This study
pMT94-MmP1	pHbPBC derivate, expressing "P_{MmP1}-$P_{porin42}$-*lysE*-P_{MmP1} (reverse)"	This study

9 **Supplementary Table 3. Relevant sequences used in this study.**

Name	Description	Sequence
MmP1 RNAP[6]	Phage RNAP from *Morganella* phage MmP1	MSIAAAVNKNDFSDVELAAIPFNTLADHYG<br>ADLAREQLQLEHESYVMGEERFRKMLERQE<br>KAEEFGDSSVAKPLIITLLPKVTQRITDWLNE<br>WADPNKKGRKPIAYTHLKDIKPETLAFITIK<br>VVLNKLAGKDDAFMQPLAYAIGSSIEDEAR<br>FGRIRELEMAHFKKCAEENLNKRRGTAYRK<br>AFLSVVEADMLDKGLLGGESWGTWNKTDV<br>MNIGISMLEKLIEATGLVELREKRNFEEMDR<br>IVIAEEYVKAMATRAQSLAGISPMYQPCVVP<br>PKPWVSITGGGYWANGRKPTALIRTHTRKA<br>LYRYEDVYMPEVYKAINYAQETPWRINRKV<br>LAVVNELVKWKNNPVKDMPSIDKLELPQRP<br>DDIDTNEEALRSWKREAAAVYRKDEQRKSR<br>YLSMSFALEQANKFSNKKAIYFPYNMDWR<br>GRVYALPMFNPQGNDMVKGLLTLAKGKPI<br>GKDGFYWLKIHGANTAGVDKVTFPERIKFIE<br>DNHDNIMQCAESPLDNLWWTEQDSPFCFLA<br>FCFEYAQVTKKGLGWVCSLPIALDGSCSGIQ<br>HFSAMLRDDIGGRAVNLLPSETVQDIYGIVA<br>DKVNEALKELVINGTDNYTDTVTDKSTGEII<br>ERYRLGEKELARQWLEFGVTRSVTKRSVMT<br>LAYGSKEYGFRDQVLEDTIRPAIDSGKGAM<br>FTNPSQAASFMAKRIWEAVSVTVVAAVGA<br>MKWLQSSAKLMAAEVKDKKTKEVLRKRC<br>AVHWVTPDGFPVWQEYRKPKQKRVHLMFL<br>GSYYDARMKETSSDCSIDAHKQESGISPNFV<br>HSQDGNHLRMTVVYAREKYNVESFALIHDS<br>FGTIPADVPNLFKAVRETMVNMYENNDVLA<br>DFYEQFADQLHESQLDKMPALPPKGKLNLQ<br>DILKSDFAFA*

K1F RNAP[6]	Phage RNAP from Enterobacteria phage K1F	MSVISIDKHDFSDVSNAIEPFNLLADHY<br>GQDLAVKQLQLEHEAYTEGERRFIKNL<br>ERQTERGELADNQVAKPLMQTLVPKIA<br>QAVKEWHEGPDGKLSTSRPSVAFTMLS<br>TEERAVKDRSLRISCESAAVIILKVILSKL<br>VKPEGIPITPMASAIGRTLEDEIRFGRIRD<br>KEKEHFKKAIADNLNKRAGASYKKAY<br>MQAVEASMLEQGQLEDAWGTWSPTEA<br>VHVGIKMLEIVIQSTQLVELKRYGAGNA<br>AADVEMVHLSDFWVKKMAQRGFSLAG<br>IAPVYQPCVVPPKPWTGVVGGGYWAK<br>GRRPLPLIRLGSKSAVARYEDVYMPEVY<br>EAVNIIQNTPWKVNKKVLDVVNMVEKL<br>NNTPIDDIPQMEPLKPEAYAGETEEELK<br>AWKKAAAGIYRREKARQSRRLSLSFIVN<br>QANKFSQFKAIWFPYNMDWRGRVYAV<br>PMFNPQGNDMQKGLLTLAVGKPIGADG<br>FKWLKVHGANCAGVDKVTFEERIKWV<br>EDNHDNIMAAAKAPMDSIEWWGKLDS<br>PFCFLAFCFEYAGVMHHGLSYSCSLPIA<br>FDGSCSGIQHFSAMLRDHIGGHAVNLTP<br>SGKVQDIYRIVSDRIEEELKVLLVNGTD<br>NEMVTHEDKKTGEITERLKLGTRELAR<br>QWLTYGMSRKVTKRSVMTLAYGSKEY<br>GFADQVYEDIVMPAIDSGSGAMFTEPSQ<br>ASRFMAKMIWEAVSVTVVAAVDAMK<br>WLQGAAKLLAAEVKDKKTGEILKPCLP<br>VHWVTPDGFPVWQEYRKKDTTRLNLM<br>FLGSFNLQPTVNKGTKKELDKHKQESGI<br>SPNFVHSQDGSHLRKTVVHTHRKYGVM<br>SFAVIHDSFGTIPADAEYLFRGVRETMV<br>ETYRDNDVLLDFYEQFEYQLHESQRDK<br>LPELPKKGKLNIEDILSSDFAFA*

VP4 RNAP[6]	Phage RNAP from Vibriophage VP4	MANVIKPQSHNFSDISAAILPFNVLADSYGEALAAEQLMLEHESYQLGEARFIKAMERQVERGEVSDNAVAKPLLDTLIPALAARITEFVEMKQRGKPHVSKGYFAMIKPESAAFIIVKTTLNILAKEESVPVQRVAMAIGGNIEDEIRFGRIRDEEIKHFKERVKPNLDKRNGFIYKKAYMEAVEAGMQDKGELNSTHEAWEKDVKFHVGIRAIEMLIEATGMVQLERKFKGIPDKDHEALHLAPEYVEKLTNRAHALAGISPMYQPMIVKPKRWTGVQGGGYWAKGRRPLNLIRVGSKRALDRYRQVDMPEVYDAINTIQETAWRINKDVLAVVNNVVTWTNCPVEDVPSIDKLALPEKPEDIDNNEESLKKWKKAAAAIYRKEKARQSRRISLEFALSQANKFSKYNEIYFPYNMDWRGRVYAIPMFNPQGNDMVKGLLTFAKKVPVGIDGGYWLAVHGANCAGVDKVSLEDRVKWVNDNEANIIASAEAPLDFTWWAEQDSPFCFLAFCFEWAAYVKAGKKPSFESSLPLAFDGTCSGLQHFSAMLRDEIGGAAVNLLPADKPQDIYGIVAVKVNEVLRDLVISGTEDEMQTLEDKKTGEITERLVLGTRTLAAQWLEYGVTRSVTKRSVMTLAYGSKEYGFADQVFEDTVMPAIDNGKGTMFTEPSQACRFMAKLIWDAVSKTVVAAVEAMQWLQSAAKLVSSEVKDKKSGEILKHAMPVHWTTPNGFPVWSEYCKQEQKVIDCVILGSMRLQLKLNMRDKKEIDTAKQASGIAPNFVHSMDASHLQMTVNKCFKVYGIHSFAMIHDSFGCHAGFASKMFRAVRETMVETYEEHDVIQEFYNQFEKQLHESQIEKMPALPRKGNLELREILKSLYTFS*
PmCDA1[7]	Cytosine deaminase with C to T activity from sea lamprey	MTDAEYVRIHEKLDIYTFKKQFFNNKKSVSHRCYVLFELKRRGERRACFWGYAVNKPQSGTERGIHAEIFSIRKVEEYLRDNPGQFTINWYSSWSPCADCAEKILEWYNQELRGNGHTLKIWACKLYYEKNARNQIGLWNLRDNGVGLNVMVSEHYQCCRKIFIQSSHNQLNENRWLEKTLKRAEKRRSELSIMIQVKILHTTKSPAV*

evoPmCDA1[8]	PmCDA1 variant with enhanced C to T activity	MTDAEYVRIHEKLDIYTFKKQFSNNKKSVSHRCYVLFELKRRGERRACFWGYAVNKPQSGTERGIHAEIFSIRKVEEYLRDNPGQFTINWYSSWSPCADCAEKILEWYNQELRGNGHTLKIWVCKLYYEKNARNQIGLWNLRDNGVGLNVMVSEHYQCCRKIFIQSSHNQLNENRWLEKTLKRAEKRRSELSIMFQVKILHTTKSPAV*
UGI[9]	Uracil glycosylase inhibitor from *Bacillus subtilis* bacteriophage PBS1	MTNLSDIIEKETGKQLVIQESILMLPEEVEEVIGNKPESDILVHTAYDESTDENVMLLTSDAPEYKPWALVIQDSNGENKIKML*
TadA7.10[10]	tRNA adenosine deaminase 7.10 with A to G activity from *E.coli*	MSEVEFSHEYWMRHALTLAKRARDEREVPVGAVLVLNNRVIGEGWNRAIGLHDPTAHAEIMALRQGGLVMQNYRLIDATLYVTFEPCVMCAGAMIHSRIGRVVFGVRNAKTGAAGSLMDVLHYPGMNHRVEITEGILADECAALLCYFFRMPRQVFNAQKKAQSSTD*
TadA8e[11]	tRNA adenosine deaminase 8e with A to G activity from *E.coli*	MSEVEFSHEYWMRHALTLAKRARDEREVPVGAVLVLNNRVIGEGWNRAIGLHDPTAHAEIMALRQGGLVMQNYRLIDATLYVTFEPCVMCAGAMIHSRIGRVVFGWRNSKRGAAGSLMNVLNYPGMNHRVEITEGILADECAALLCDFYRMPRQVFNAQKKAQSSIN*
TadA9[12]	tRNA adenosine deaminase 9 with A to G activity from *E.coli*	MSEVEFSHEYWMRHALTLAKRARDEREVPVGAVLVLNNRVIGEGWNRAIGLHDPTAHAEIMALRQGGLVMQNYRLIDATLYSTFEPCVMCAGAMIHSRIGRVVFGWRNSKRGAAGSLMNVLNYPGMNHRVEITEGILADECAALLCDFYRMPRRVFNAQKKAQSSIN*
CABE T3.1[13]	TadA variant, dual deaminase with both C to T and A to G activity	MSEVEFSHEYWMRHALTLAKRARDERSVPVGAVLVLNNRVIGEGWNRAKGLHDPTAHAEIMALRQGGLVMQNYRLYDATLYTTFEPCVMCAGAMIHSRIGRVVFGVRNAKTGAAGSLMDVLHHPGMNHRVEITEGILADECAALLCRFFRMPRRVFNAQKKAQSSTD*
CABE T3.155[13]	TadA variant, dual deaminase with both C to T and A to G activity	MSEVEFSHEYWMRHALTLAKRARDERSVPVGAVLVLNNRVIGEGWNRAIGLHDPTAHAEIMALRQGGLVMQNYRLYDATLYTTFEPCVMCAGAMIHSRIGRVVFGVRNAKTGAAGSLMDVLHHPGMNHRVEITEGILADECAALLCRFFRMPRRVFNAQKK

		AQSSTD*
TadDE[14]	TadA variant, dual deaminase with both C to T and A to G activity	MSEVEFSHEYWMRHALTLAKRARDEGEAPVGAVLVLNNRVIGEGWNRRIGLHDPTAHAEIMALRQGGLVMQNSRLIDATLYVTFEPCVMCAGAMINSRIGRVVFGVRNSKRGAAGSLMNVLNYPGMNHRVEITEGILADECAALLCDFYRMPRQVFNAQKKAQSSIN*
P_{MmP1}[6]	promoter of MmP1 RNAP	cccatgagttaattatatttgtggcattataggg
P_{K1F}[6]	promoter of K1F RNAP	gacatggctcaagcctaaactatcactatagg
P_{VP4}[6]	promoter of VP4 RNAP	gaagtaacttgattaattaaccctgactataggga

Supplementary Table 4. Mutation summary for fluorescent protein mutants.

Strains	mCherry	sfGFP	TagBFP
Mutant1	F14F(TTC-TTT)	Q184*(CAA-TAA), D190N(GAT-AAT)	R179K(AGA-AAA)
Mutant2	V7V(GTG-GTA), Y120Y(TAC-TAT)	-	L4L(CTG-CTA), V132M(GTG-ATG), M160I(ATG-ATA), L170L(CTG-CTA), R179K(AGA-AAA)
Mutant3	-	G104D(GGC-GAC), H148Y(CAC-TAC)	-
Mutant4	F99F(TTC-TTT)	-	E29K(AGAG-AAG), R220K(AGA-AAA)

Supplementary Table 5. Mutation summary for chromoprotein mutants.

	amajLime	fwYellow	spisPink
Mutant1	E32K(GAA-AAA), D61N(GAT-AAT), D134N(GAT-AAT), P147P(CCG-GGA), P188P(CCG-GGA), D205N(GAT-AAT), L206L(CTG-CTA), D207N(GAC-AAC), G210S(GGC-AGC), V213I(GTT-ATT), S225S(TCG-TCA)	A40A(GCG-GCA)	S4L(TCA-TTA), F40F(TTC-TTT)
Mutant2	M12I(ATG-ATA), D103N(GAT-AAT), L159L(CTG-CTA)	E22K(GAA-AAA)	-
Mutant3	M12I(ATG-ATA), R72H(CGT-CAT), D103N(GAT-AAT)	E22K(GAA-AAA)	-
Mutant4	D10N(GAT-AAT), D11N(GAT-AAT), V29V(GTG-GTA), T49T(ACG-ACA), S110T(AGC-AAC), W111*(TGG-TAG), T216T(ACG-ACA)	I227I(ATC-ATT)	P37L(CCG-CTG), F71F(TTC-TTT), I157I(ATC-ATT), P186L(CCA-CTA), L213L(CTG-TTG)
Mutant5	Q87*(CAA-TAA), D134I(GAT-AAT), P136S(CCG-TCG)	A8V(GCA-GTA), A111A(GCG-GCA), P190P(CCG-CCA)	L156L(CTG-CTA), G169G(GGC-GGT)
Mutant6	E121K(GAA-AAA), G135S(GGT-AGT)	V226V(GTG-GTA)	-
Mutant7	L3L(CTG-CTA)	-	T34I(ACC-ATC), L65L(CTG-TTG), Y93Y(TAC-TAT)
Mutant8	G35S(GGT-AGT), L68L(CTG-CTA), P78P(CCG-CCA), G92S(GGC-AGC), D103N(GAT-AAT), G104S(GGC-AGC), C119Y(TGT-TAT), E121K(GAA-AAA), R200H(CGT-CAT)	E91K(GAA-AAA), E116K(GAA-AAA), V226V(GTG-GTA)	D10N(GAT-AAT),D78N(GAT-AAT)

Supplementary Table 6. Mutation summary for *ftsQAZ* mutants in FtsQ, FtsA, and FtsZ.

	FtsQ	FtsA	FtsZ
ftsQAZ mutant1	-	-	L125L(CTG-CTA), T267A(ACT-GCT), D306G(GAT-GGT), R357R(CGT-CGC)
ftsQAZ mutant2	-	-	I266V(ATT-GTT)
ftsQAZ mutant3	-	-	K153R(AAA-AGA), I266V(ATT-GTT)
ftsQAZ mutant4	-	-	I266V(ATT-GTT), T348T(ACT-ACC)
ftsQAZ mutant5	R192Q(CGA-CAA)	-	-
ftsQAZ mutant6		R177Q(CGG-CAG)	E252K(GAA-AAA)

Supplementary Table 7. Mutation summary for *mreBCD* mutants in MreB, MreC, and MreD.

	MreB	MreC	MreD
mreBCD mutant1	-	T282A(ACT-GCT)	W31R(TGG-CGG)
mreBCD mutant2	-	-	L121S(TTA-TCA)
mreBCD mutant3	-	V195A(GTG-GCG)	-
mreBCD mutant4	G165G(GGT-GGC)	L159L(TTA-TTG), V187A(GTG-GCG), T213T(ACC-ACT), I301I(ATC-ATT)	L65L(CTG-CTA)

Supplementary Table 8. Mutation summary for *ftsQAZ&mreBCD* mutants in FtsQ, FtsA, and FtsZ.

	FtsQ	FtsA	FtsZ
ftsQAZ&mreBCD mutant1	-	-	-
ftsQAZ&mreBCD mutant2	-	-	E150K(GAA-AAA), S333S(TCT-TCC)
ftsQAZ&mreBCD mutant3	-	I352I(ATT-ATC)	D255D(GAT-GAC), P369P(CCT-CCC)
ftsQAZ&mreBCD mutant4	-	-	S299S(TCT-TCC)

Supplementary Table 9. Mutation summary for *ftsQAZ&mreBCD* in MreB, MreC, and MreD.

	MreB	MreC	MreD
ftsQAZ&mreBCD mutant1	-	G65G(GGT-GGC), V138A(GTG-GCG)	V114A(GTG-GCG)
ftsQAZ&mreBCD mutant2	-	Q136R(CAG-CGG)	F14F(TTT-TTC), V33A(GTA-GCA), V61A(GTA-GCA)
ftsQAZ&mreBCD mutant3	-	D71D(GAT-GAC)	V114A(GTG-GCG)
ftsQAZ&mreBCD mutant4	-	-	L22L(TTA-TTG), Q103R(CAA-CGA), T123A(ACA-GCA)

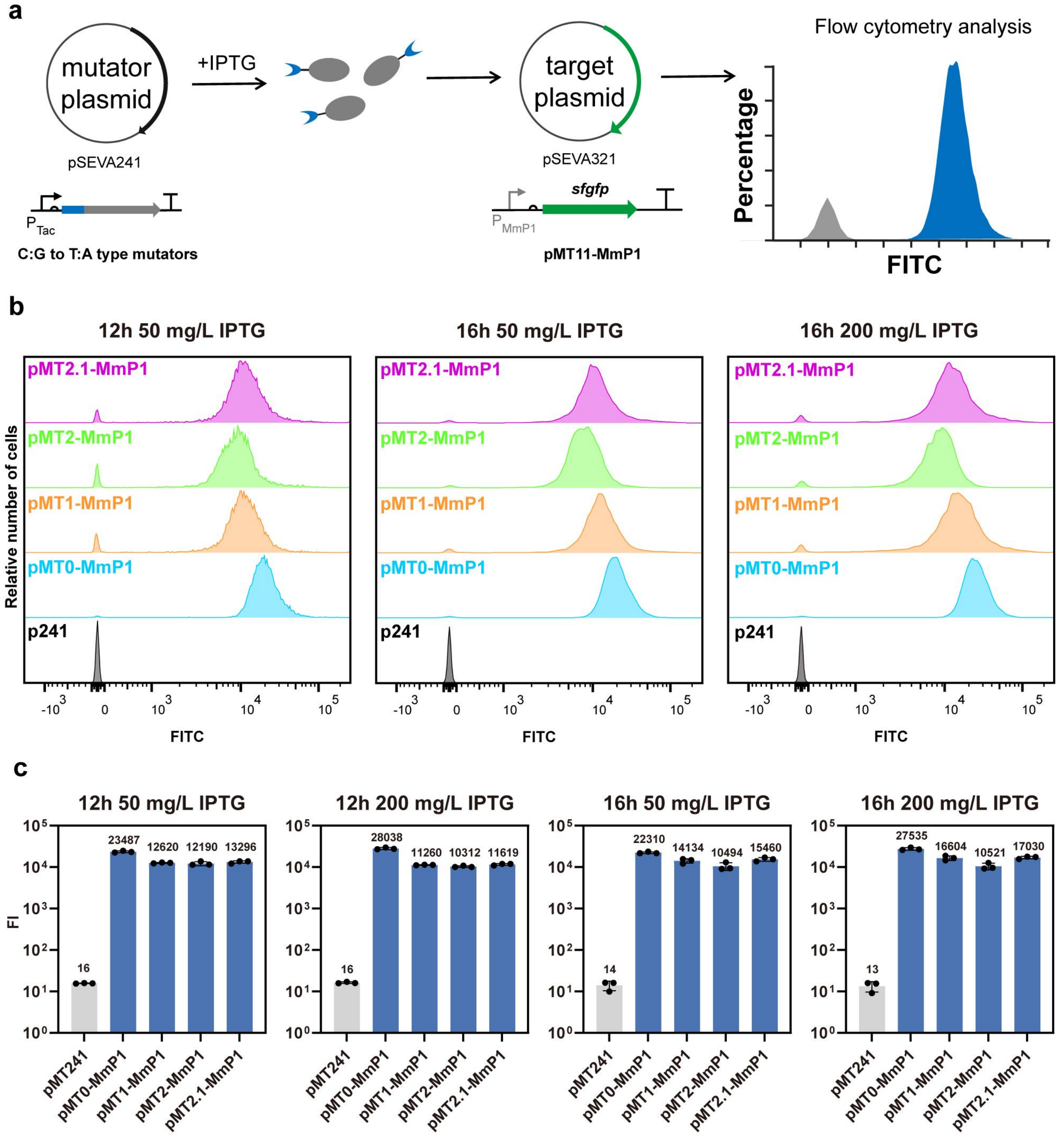

Supplementary Fig. 1 Transcriptional activity analysis of C:G to T:A type single mutators. **a** Scheme of the transcriptional activity analysis of C:G to T:A type single mutators using sfGFP as the reporter protein. **b** Analysis of the transcriptional activity of C:G to T:A type single mutators via flow cytometry under varying inducer durations (12 h or 16 h) and concentrations (50 mg/L or 200 mg/L). **c** Fluorescence intensity (FI) of sfGFP driven by various C:G to T:A type single mutators. Error bars indicate standard errors, black dots represent individual data points. *n* = 3, which represents three independent replicates of the experiment.

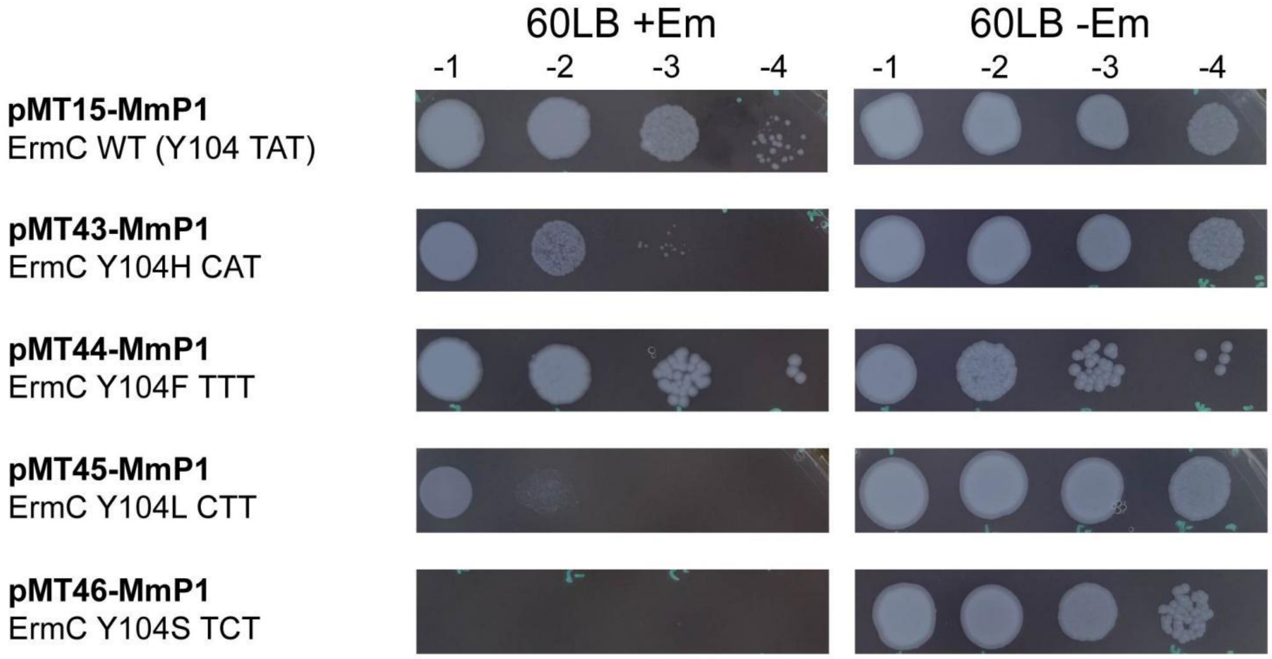

Supplementary Fig. 2 Characterization of ErmC mutants for assessment of C:G to T:A type mutation rates in *H. bluephagenesis* TD01.

Cell viability of various ErmC mutants was evaluated in 60LB medium, with or without 200 mg/L erythromycin (Em). The culture solution was serially diluted in 10-fold steps with 60LB medium, and 10 μL of each dilution was placed on solid agar plates.

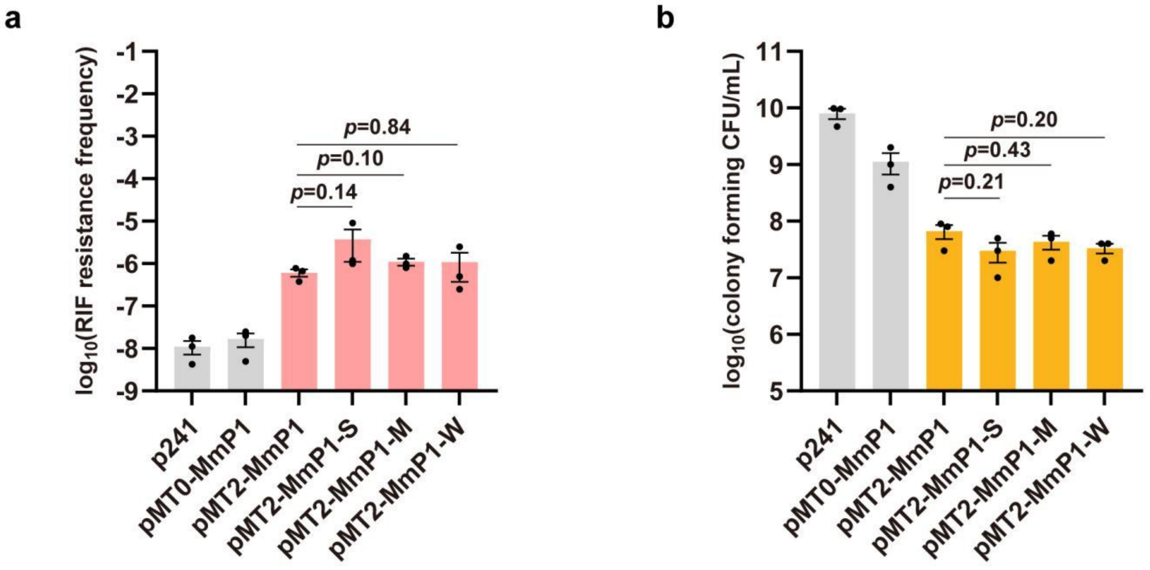

Supplementary Fig. 3 The off-target effect and cell viability analysis of PmCDA1 mutators with three different strengths of RBS.

a Analysis of off-target mutation rate for PmCDA1 mutators with three different strengths of RBS using rifampicin resistance frequency. **b** Analysis of colony formation for PmCDA1 mutators with three different strengths of RBS. Bars, error bars, and black dots represent mean values, standard errors, and individual values, respectively. $n = 3$, which represents three independent replicates of the experiment. Statistical analyses were conducted using two-tailed Student's t-tests. A p value < 0.05 was considered significant. Source data are provided as a Source Data file.

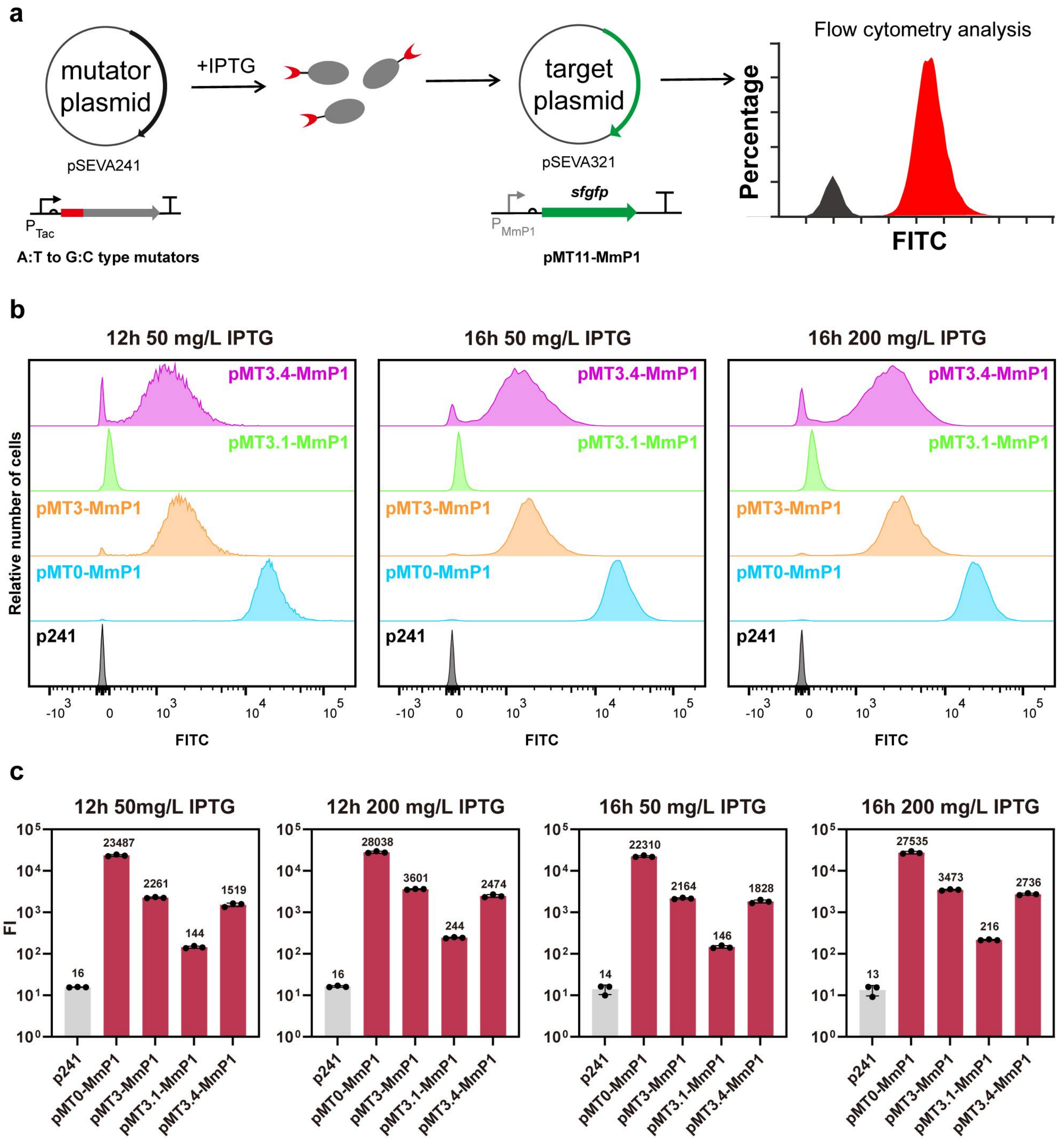

Supplementary Fig. 4 Transcriptional activity analysis of A:T to G:C type single mutators. **a** Scheme of transcriptional activity analysis of A:T to G:C type single mutators using sfGFP as the reporter protein. **b** Transcriptional activity of A:T to G:C type single mutators analyzed by flow cytometry for varying inducer durations (12 h or 16 h) and concentrations (50 mg/L or 200 mg/L). **c** Fluorescence intensity of sfGFP driven by various A:T to G:C type single mutators. Error bars indicate standard errors, black dots represent individual values. *n* = 3, which represents three independent replicates of the experiment.

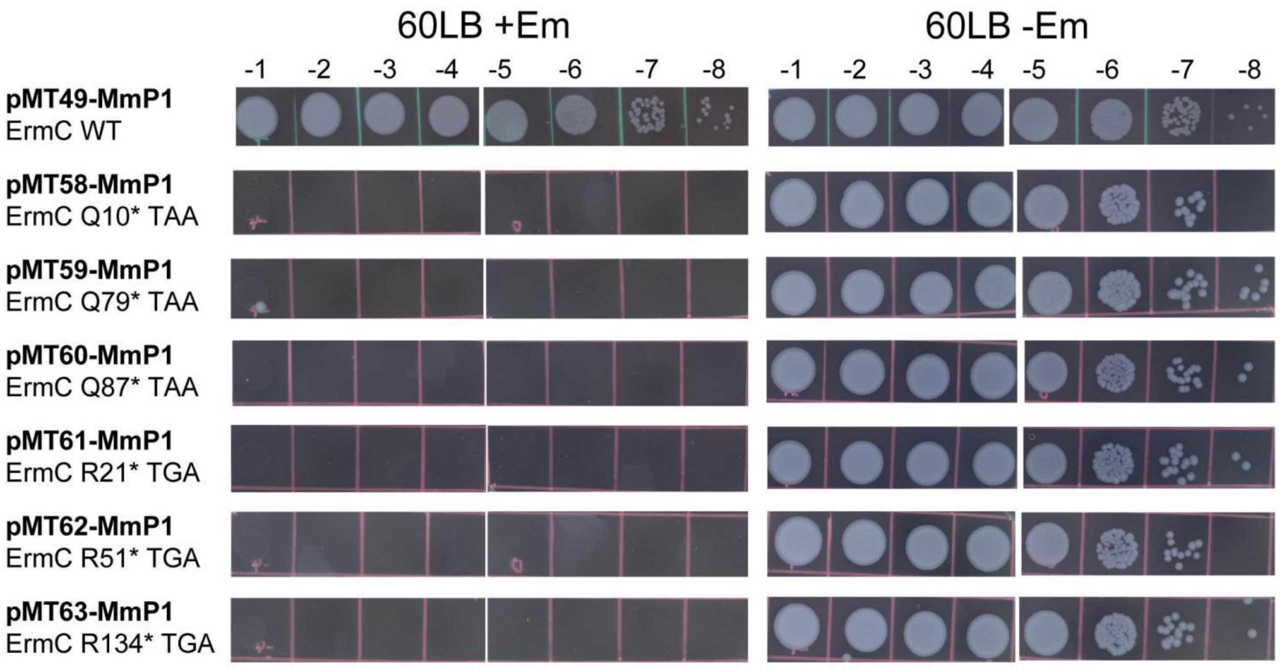

Supplementary Fig. 5 Characterization of ErmC mutants for assessing A:T to G:C type mutation rates in *H. bluephagenesis* TD01.

Cell viability of different ErmC mutants in 60LB medium, with or without 200 mg/L Em. The culture solution was serially diluted in 10-fold steps using the 60LB medium, and 10 μL of each dilution was placed on solid agar plates.

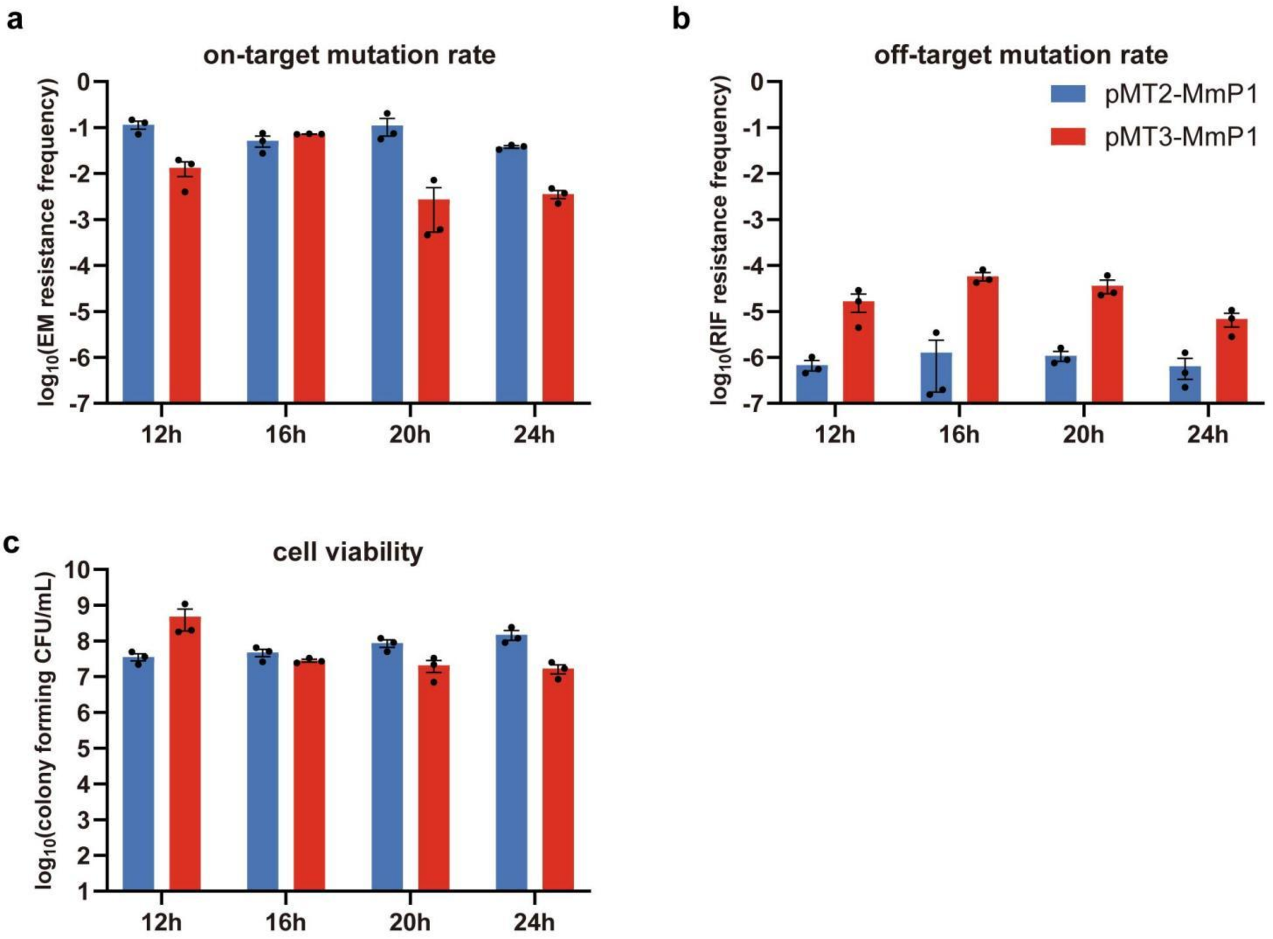

Supplementary Fig. 6 Characterization of the impact of varying induction durations on C:G to T:A and A:T to G:C type single mutators in *H. bluephagenesis*, respectively.

Analysis of on-target (**a**), off-target (**b**) mutation rates, and cell viability (**c**) of pMT2-MmP1 and pMT3-MmP1 mutators under varying inducer durations (12, 16, 20, and 24 h) with 200 mg/L IPTG. $n = 3$, which represents three independent replicates of the experiment. Source data are provided as a Source Data file.

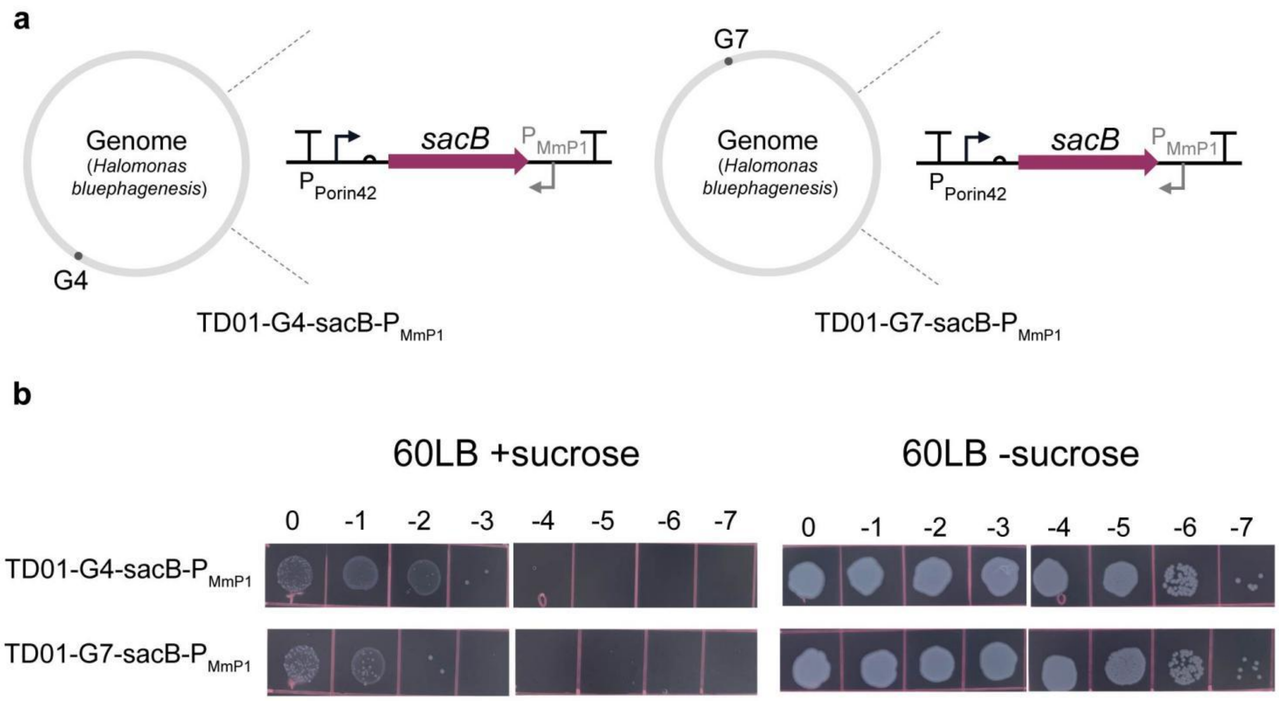

Supplementary Fig. 7 Construction and characterization of *sacB*-based reporter strains.
a Scheme of the *sacB*-based reporter strains *H. bluephagenesis* TD01-G4-*sacB*-P_{MmP1} and TD01-G7-*sacB*-P_{MmP1}. The *sacB* gene from *Bacillus subtilis* was regulated by the constitutive $P_{porin42}$ promoter with a P_{MmP1} promoter positioned downstream of the gene in the reverse orientation to recruit mutators. The reporter module was integrated at the G4 or G7 genomic locus of *H. bluephagenesis* TD01. **b** Cell viability of the two reporter strains in 60LB medium, with or without 100 g/L sucrose. The culture solution was serially diluted in 10-fold steps with 60LB medium, and 10 μL of each dilution was placed on solid agar plates.

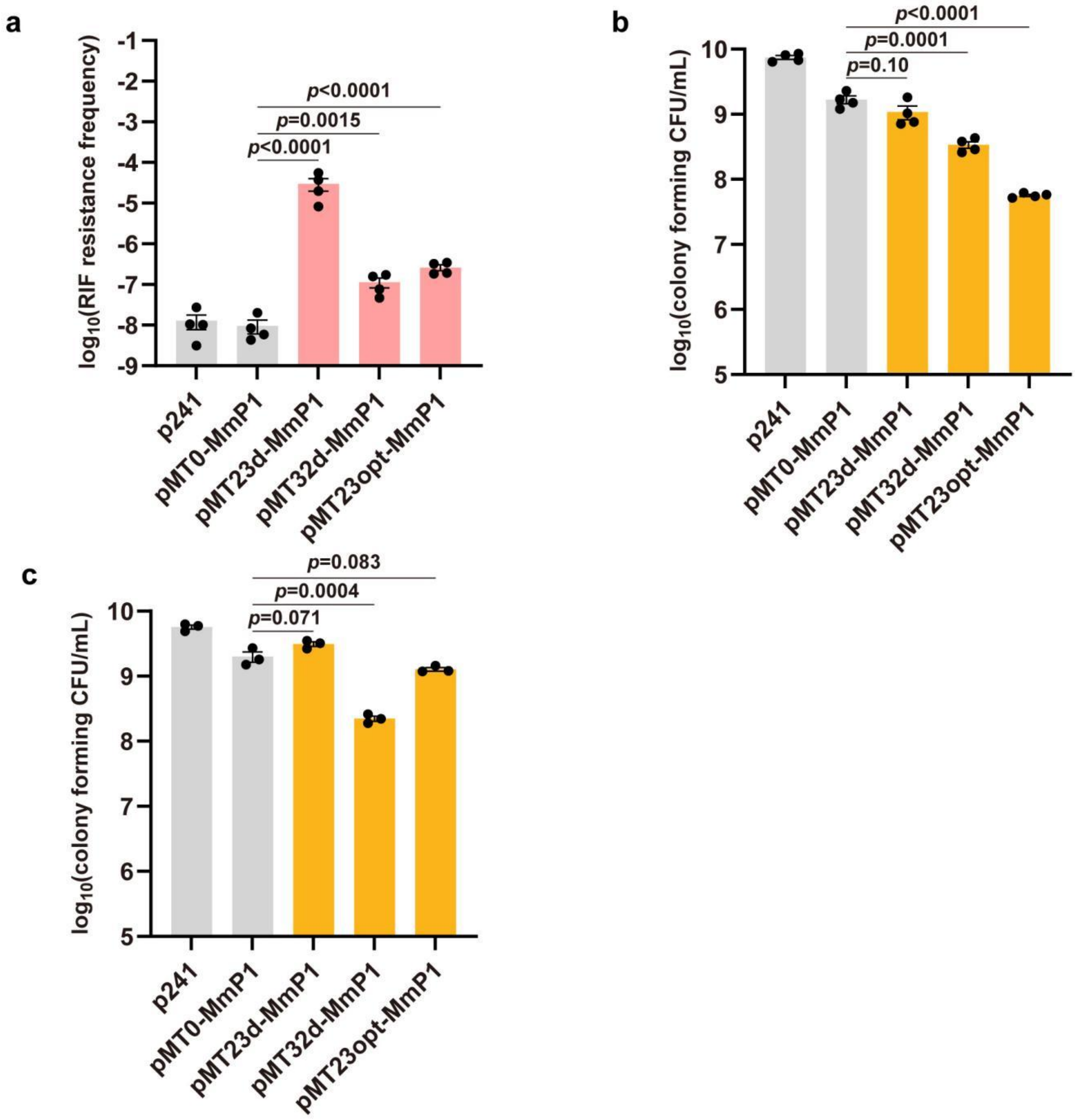

Supplementary Fig. 8 Analysis of off-target effect and cell viability of MmP1-based dual mutators.

a The rifampicin resistance frequency of MmP1-based dual mutators using *H. bluephagenesis* TD01-G7-*sacB*-P_{MmP1} reporter strain (n = 4 independent experiments). **b** Colony forming study of MmP1-based dual mutators using *H. bluephagenesis* TD01-G7-*sacB*-P_{MmP1} reporter strain (n = 4 independent experiments). **c** Colony forming analysis of MmP1-based dual mutators using *H. bluephagenesis* TD01-G7- P_{MmP1}-*sacB*-P_{MmP1} reporter strain (n = 3 independent experiments). Bars, error bars, and black dots represent mean values, standard errors, and individual values, respectively. Statistical analyses were conducted using two-tailed Student's t-tests. A p value < 0.05 was considered significant. Source data are provided as a Source Data file.

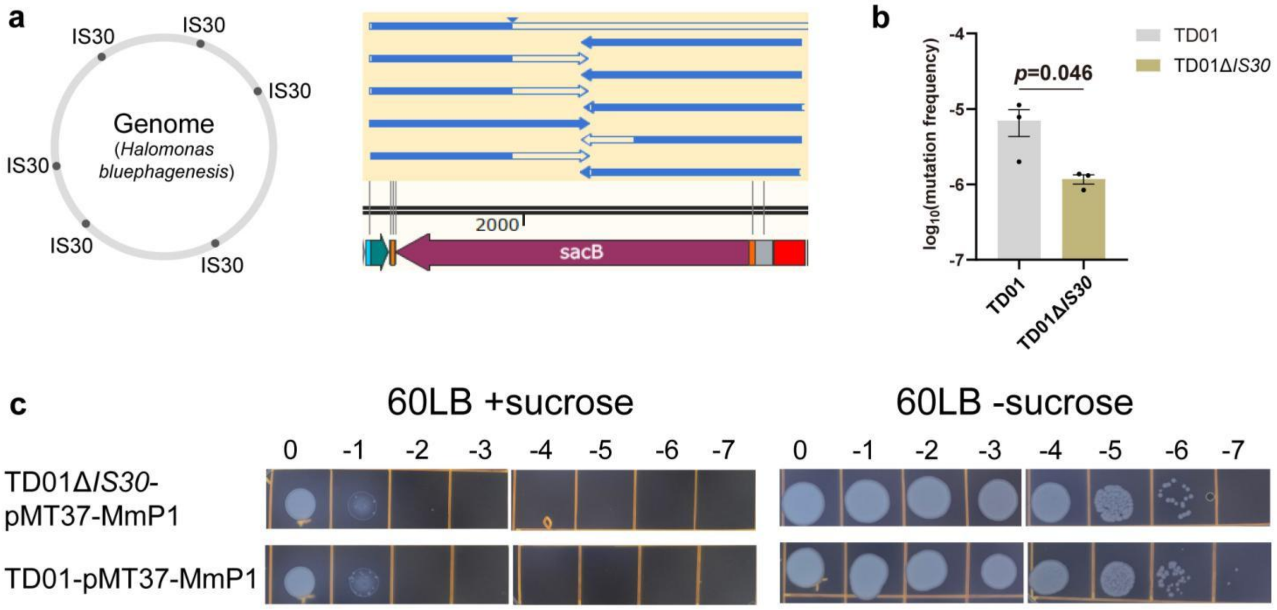

Supplementary Fig. 9 Genetic instability resulted from six IS30 family transposons in *H. bluephagenesis*

a Insertion of six IS30 family transposases in the genome inactivated SacB. **b** Deletion of IS30 transposases reduces the background mutation rate of SacB (n = 3 independent experiments). **c** Cell viability analysis of two strains in 60LB medium with or without 100 g/L sucrose. The culture solution was serially diluted in 10-fold steps with 60LB medium, and 10 μL of each dilution was placed onto solid agar plates. Bars, error bars, and black dots represent mean values, standard errors, and individual values, respectively. Statistical analyses were conducted using two-tailed Student's t-tests. A p value < 0.05 was considered significant. Source data are provided as a Source Data file.

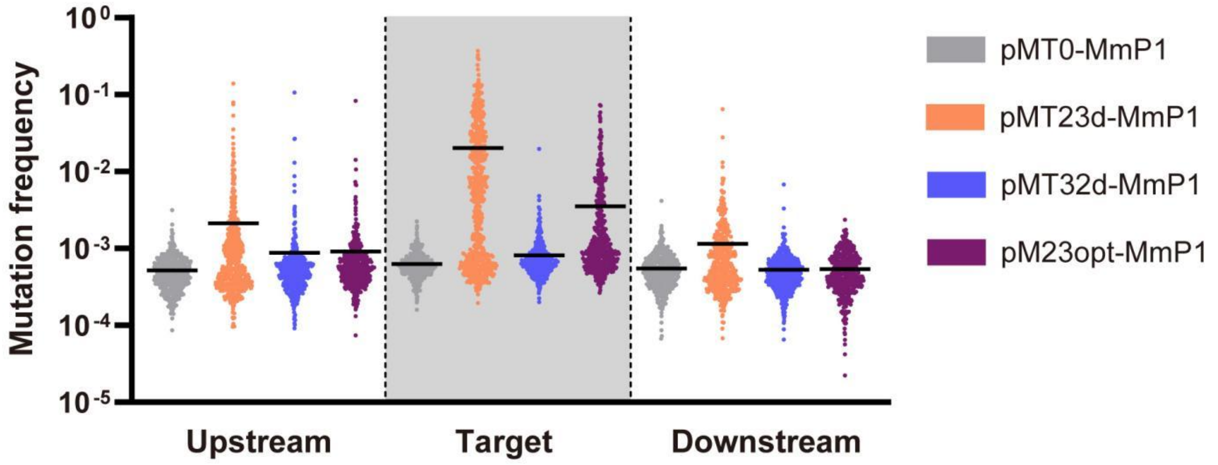

Supplementary Fig. 10 Mutation frequencies in upstream, target, and downstream regions in *H. bluephagenesis*

The mutation frequencies of three orthogonal transcription mutators and the control in upstream, target, and downstream regions are demonstrated as dot plots with mean values using black lines (ca. 10^5 reads).

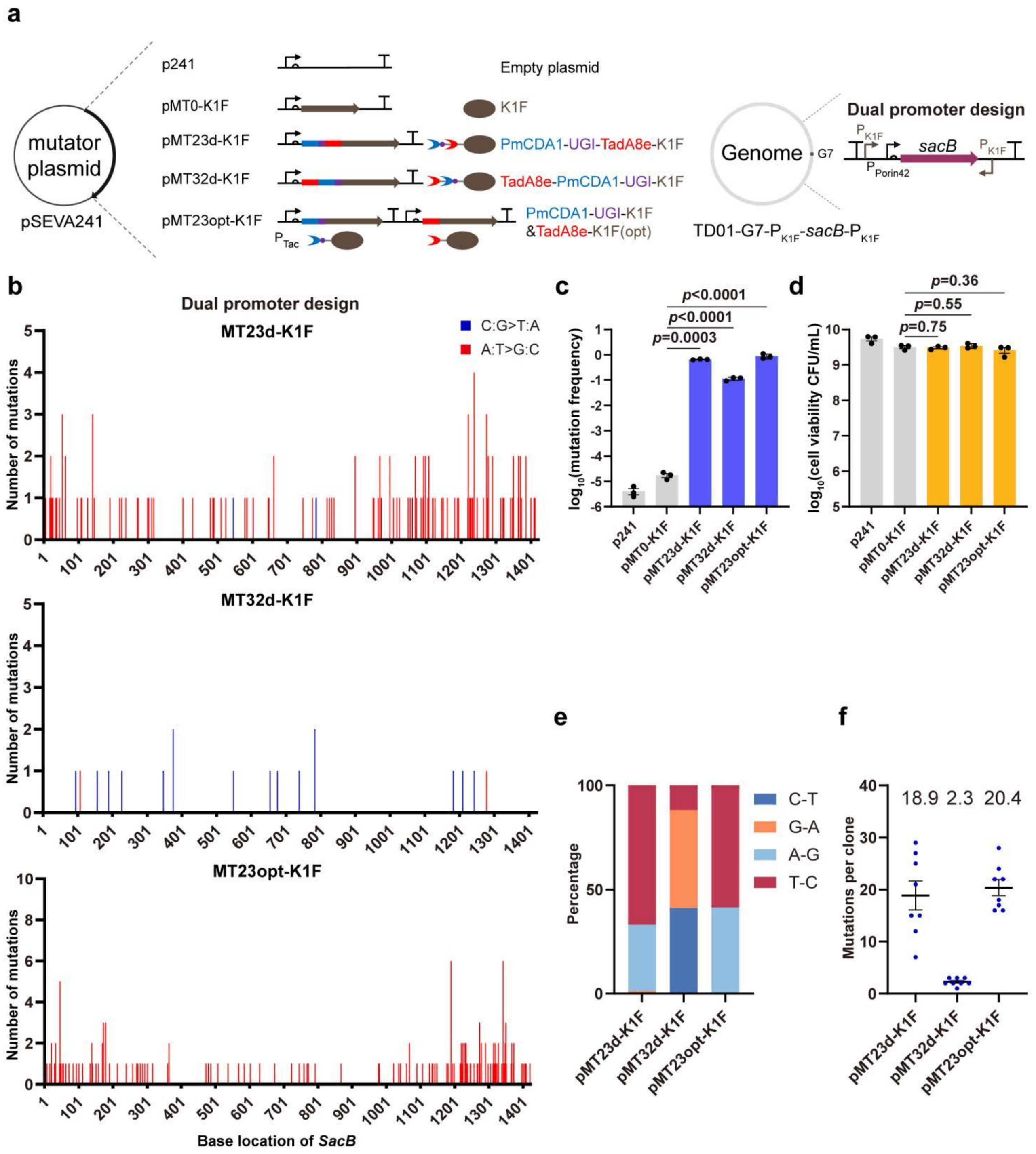

Supplementary Fig. 11 Construction and characterization of K1F-based dual mutators in *H. bluephagenesis*.

a Construction of three types of dual mutators based on K1F RNAP and the reporter strain *H. bluephagenesis* TD01-G7-P$_{K1F}$-*sacB*-P$_{K1F}$. Dual mutators were designed by fusing PmCDA1-UGI and TadA8e to K1F in two different configurations or co-expressing PmCDA1-UGI-K1F and

TadA8e-K1F (opt) in the same plasmid. The term "opt" refers to codon optimization. The “P_{K1F}-$P_{Porin42}$-*sacB*-P_{K1F} (reverse)” module was integrated into the G7 locus of the chromosome to construct the reporter strain *H. bluephagenesis* TD01-G7-P_{K1F}-*sacB*-P_{K1F}**.** **b** Distribution of mutations in the *sacB* gene using three different dual mutators. **c,d** Analysis of *sacB* mutation frequency and cell viability analysis for three dual mutators (n = 3 independent experiments). **e,f** mutation types and average number of mutations (n = 8 independent experiments) in *sacB*. Data are presented as mean values (bars), standard errors (error bars), and individual values (black or blue dots). Statistical analyses were conducted using two-tailed Student’s t-tests. A p value < 0.05 was considered significant. Source data are provided as a Source Data file.

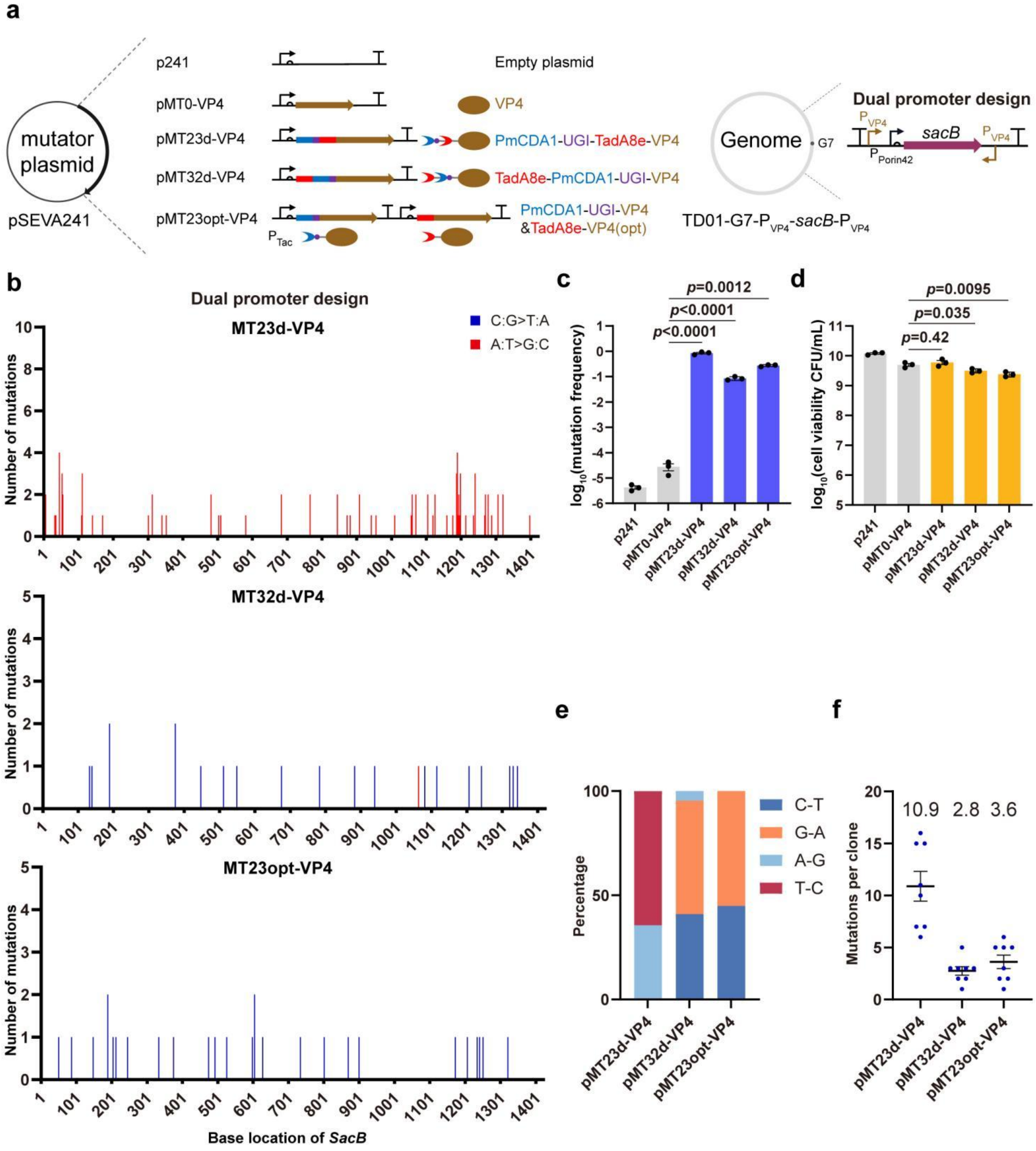

Supplementary Fig. 12 Construction and characterization of VP4-based dual mutators in *H. bluephagenesis*.

a Construction of three types of dual mutators based on VP4 RNAP and the reporter strain *H. bluephagenesis* TD01-G7-P_{VP4}-*sacB*-P_{VP4}. Dual mutators were designed by fusing PmCDA1-UGI and TadA8e to VP4 in two different configurations or by co-expressing PmCDA1-UGI-VP4 and

TadA8e-VP4 (opt) within the same plasmid. The term "opt" refers to codon optimization. The "P_{VP4}-$P_{Porin42}$-*sacB*-P_{VP4} (reverse)" module was integrated into the G7 locus to construct the reporter strain *H. bluephagenesis* TD01-G7-P $_{VP4}$-*sacB*-P $_{VP4}$. **b** Distribution of mutations in the *sacB* gene using three different dual mutators. **c,d** Analysis of *sacB* mutation frequency and cell viability for three dual mutators (n = 3 independent experiments). **e,f** Mutation types and average number of mutations (n = 8 independent experiments) in *sacB*. Data are presented as mean values (bars), standard errors (error bars), and individual values (black or blue dots). Statistical analyses were conducted using two-tailed Student's t-tests. A p value < 0.05 was considered significant. Source data are provided as a Source Data file.

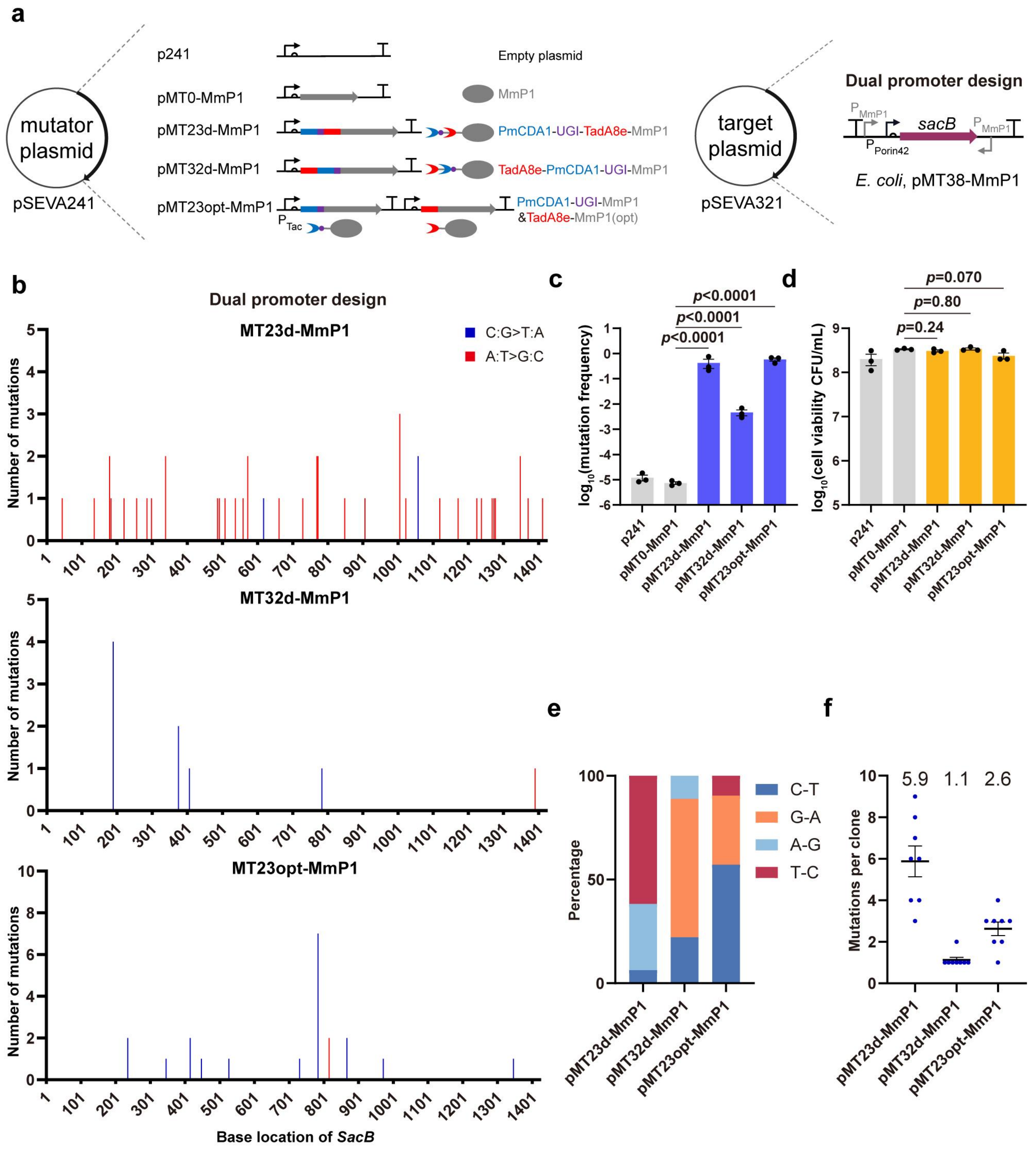

Supplementary Fig. 13 Expansion of MmP1-based dual mutators into *E.coli*.

a Three types of dual mutators based on MmP1 RNAP and the reporter strain *E. coli* MG1655, containing the target plasmid pMT38-MmP1, expressing "P_{MmP1}-$P_{Porin42}$-*sacB*-P_{MmP1} (reverse)" module. **b** Distribution of mutations in the *sacB* gene using three different dual mutators in *E. coli*. **c,d** Analysis of *sacB* mutation frequency and cell viability for three dual mutators (*n* = 3

independent experiments). **e,f** Mutation types and average number of mutations (n = 8 independent experiments) in *sacB*. Data are presented as mean values (bars), standard errors (error bars), and individual values (black or blue dots). Statistical analyses were conducted using two-tailed Student's t-tests. A p value < 0.05 was considered significant. Source data are provided as a Source Data file.

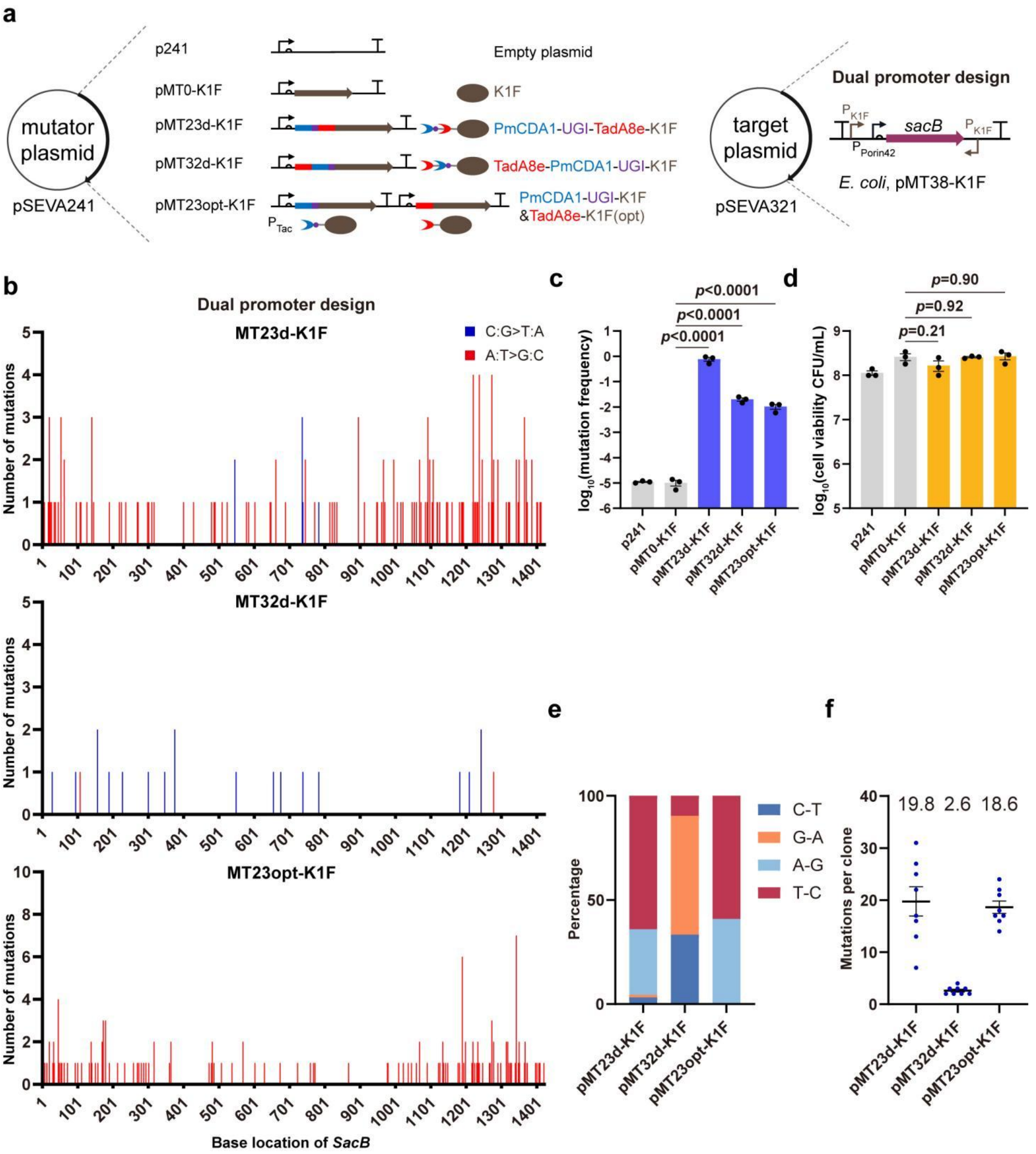

Supplementary Fig. 14 Expansion of K1F-based dual mutators into *E. coli*.

a Three types of dual mutators based on K1F RNAP and the reporter strain *E. coli* MG1655, containing the target plasmid pMT38-K1F, expressing "P_{K1F}-$P_{Porin42}$-*sacB*-P_{K1F} (reverse)" module. **b** Distribution of mutations in the *sacB* gene using three different dual mutators in *E. coli*. **c,d** Analysis of *sacB* mutation frequency and cell viability for three dual mutators (n = 3 independent experiments). **e,f** Mutation types and average number of mutations (n = 8 independent experiments) in *sacB*. Data are presented as mean values (bars), standard errors (error bars), and individual values

(black or blue dots). Statistical analyses were conducted using two-tailed Student's t-tests. A p value < 0.05 was considered significant. Source data are provided as a Source Data file.

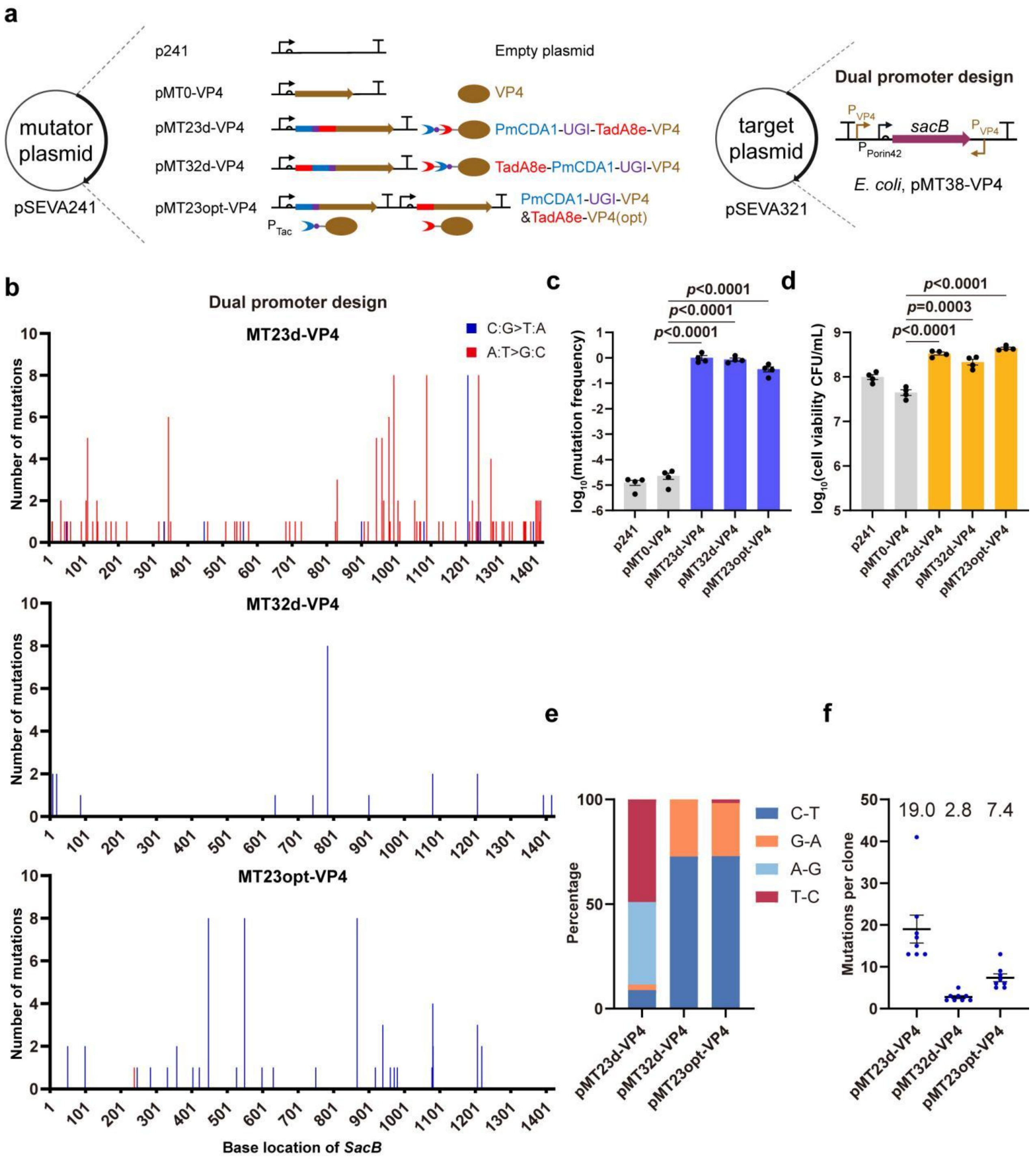

Supplementary Fig. 15 Expansion of VP4-based dual mutators into *E. coli*.

a Three types of dual mutators based on VP4 RNAP and the reporter strain *E. coli* MG1655, containing the target plasmid pMT38-VP4, expressing "P_{VP4}-$P_{Porin42}$-*sacB*-P_{VP4} (reverse)" module. **b** Distribution of mutations in the *sacB* gene using three different dual mutators in *E. coli*. **c,d** Analysis of *sacB* mutation frequency and cell viability for three dual mutators (*n* = 3 independent experiments). **e,f** Mutation types and average number of mutations (*n* = 8 independent experiments) in *sacB*. Data are presented as mean values (bars), standard errors (error bars), and individual values

(black or blue dots). Statistical analyses were conducted using two-tailed Student's t-tests. A p value < 0.05 was considered significant. Source data are provided as a Source Data file.

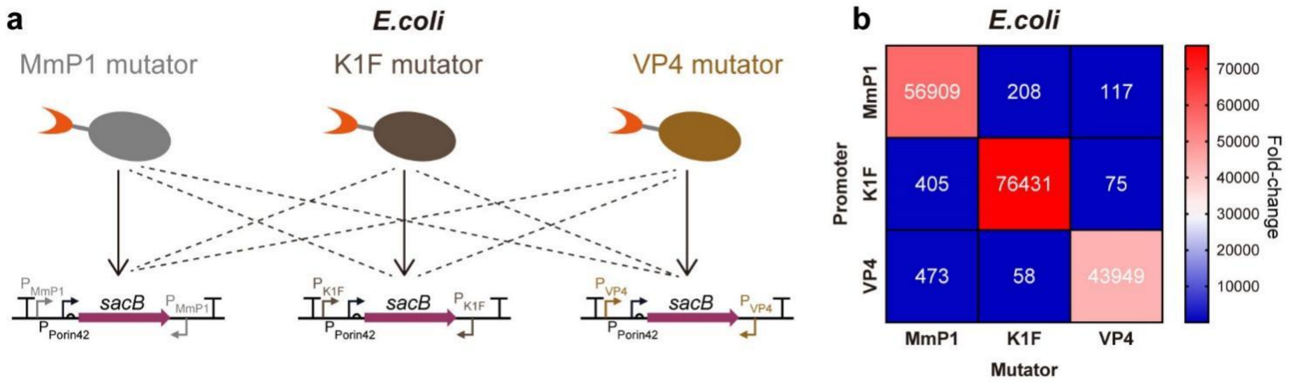

Supplementary Fig. 16 Orthogonality of dual mutators based on three phage RNAPs in *E. coli*. **a** Scheme of orthogonality of dual mutators based on three phage RNAPs in *E. coli*. **b** Heat map profiling of fold-change in mutation rate by three dual mutators in *E. coli*. $n = 3$, which represents three independent replicates of the experiment.

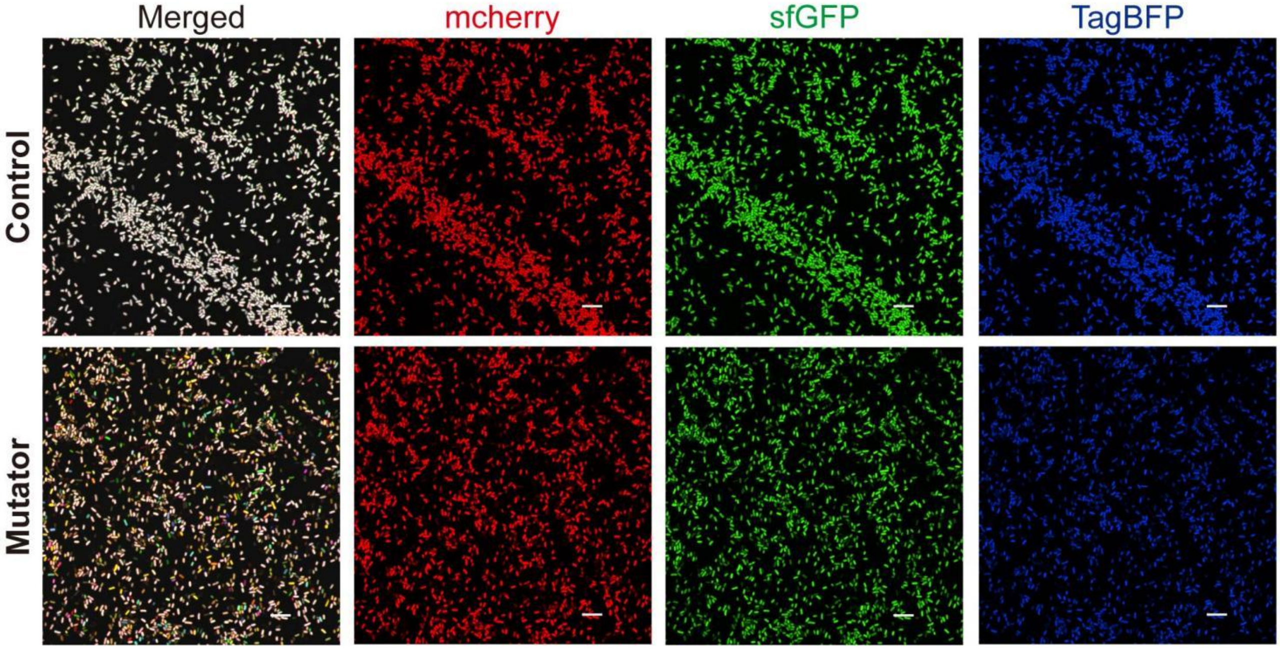

Supplementary Fig. 17 Mutagenesis of fluorescent proteins generates colorful cells of *E. coli*. Confocal microscopy analysis of color changes in the control (pMT0-MmP1) and mutator groups (pMT23opt-MmP1) in 10LB medium from the green, red, and blue channels. Scale bar = 10 μm. The experiment was repeated three times with similar results.

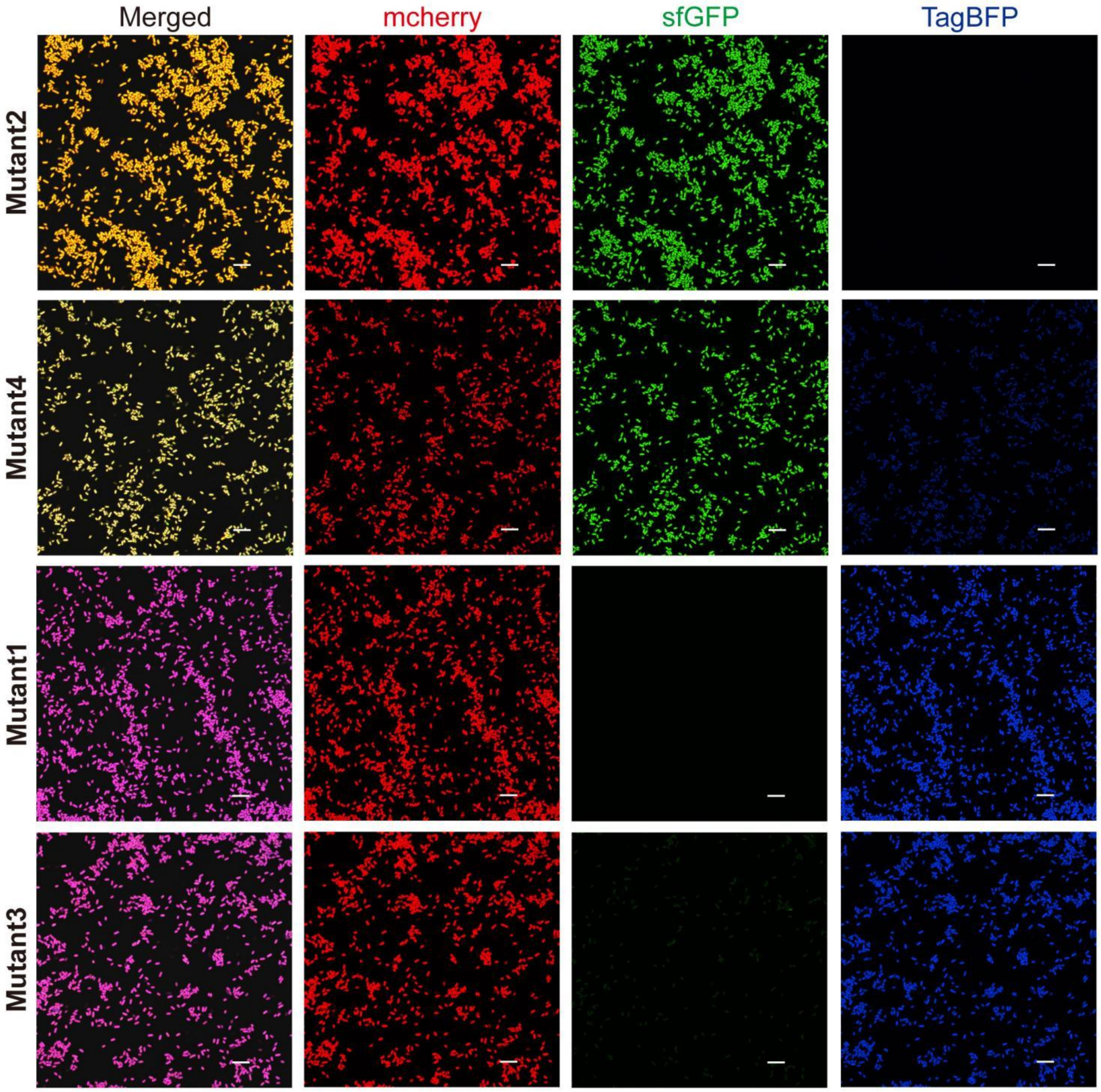

Supplementary Fig. 18 Confocal microscopy analysis of fluorescent protein mutants in *E. coli*.

Confocal microscopy analysis of four fluorescent protein mutants in 10LB medium from the green, red, and blue channels. Scale bar = 10 μm. The experiment was repeated three times with similar results.

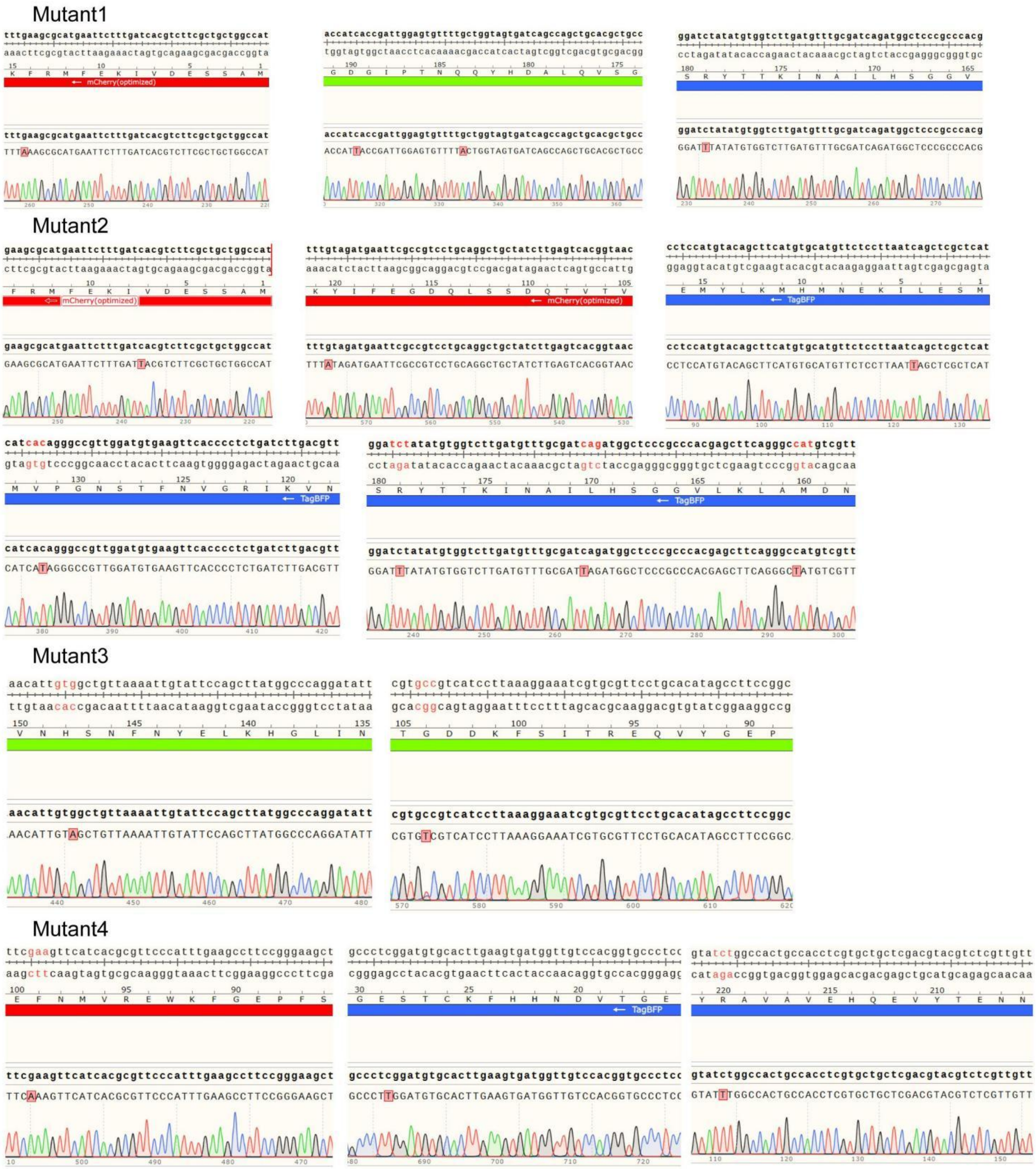

Supplementary Fig. 19 Sequencing of fluorescent protein mutants in *E. coli*.

Sequencing of four fluorescent protein mutants in mCherry, sfGFP, and TagBFP, respectively.

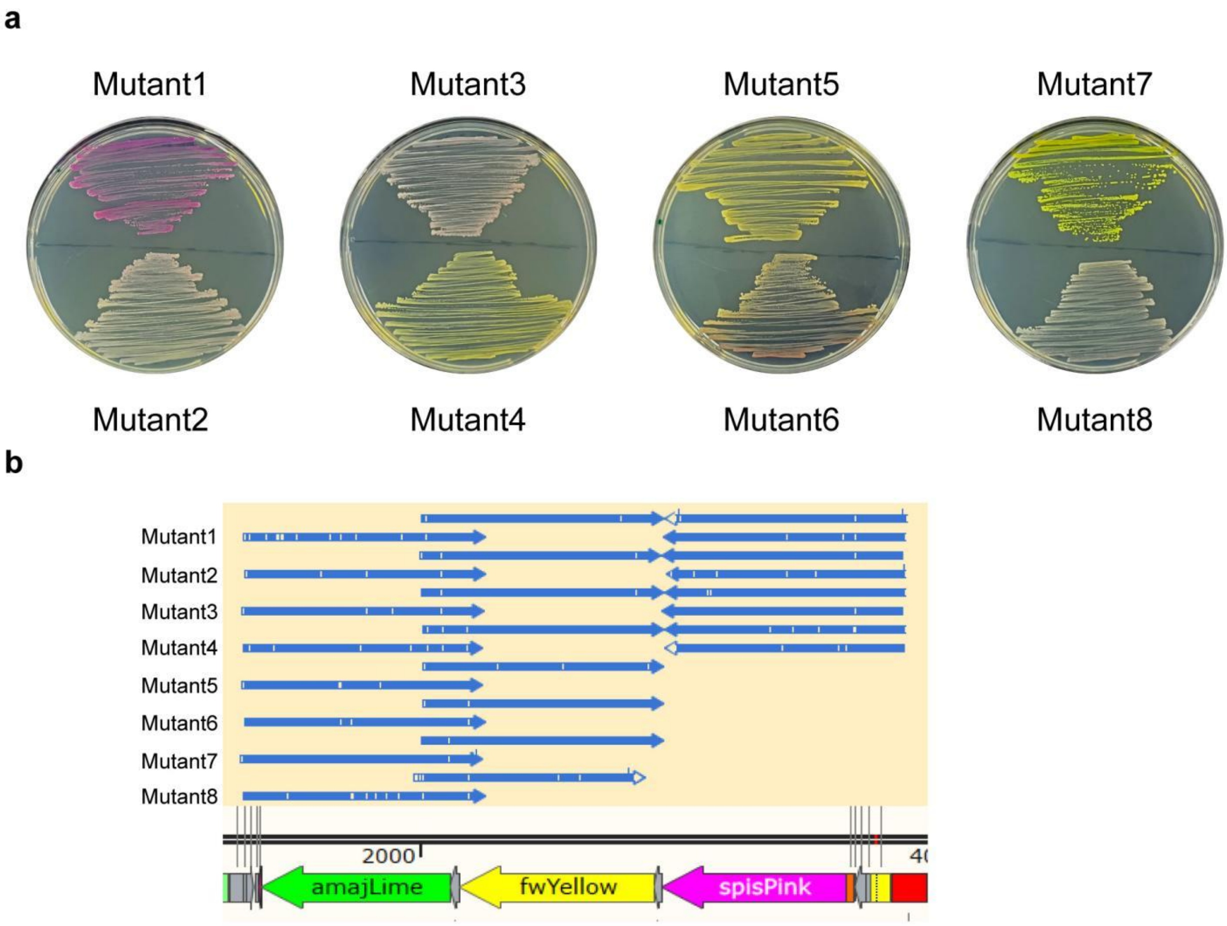

Supplementary Fig. 20 Colors and sequencing of chromoprotein mutants in *E. coli*.

a Eight chromoprotein mutants with distant color changes were selected after mutagenesis and streaked on 10LB agar plates. **b** Sequencing of eight chromoprotein mutants in amajLime, fwYellow, and spisPink, respectively.

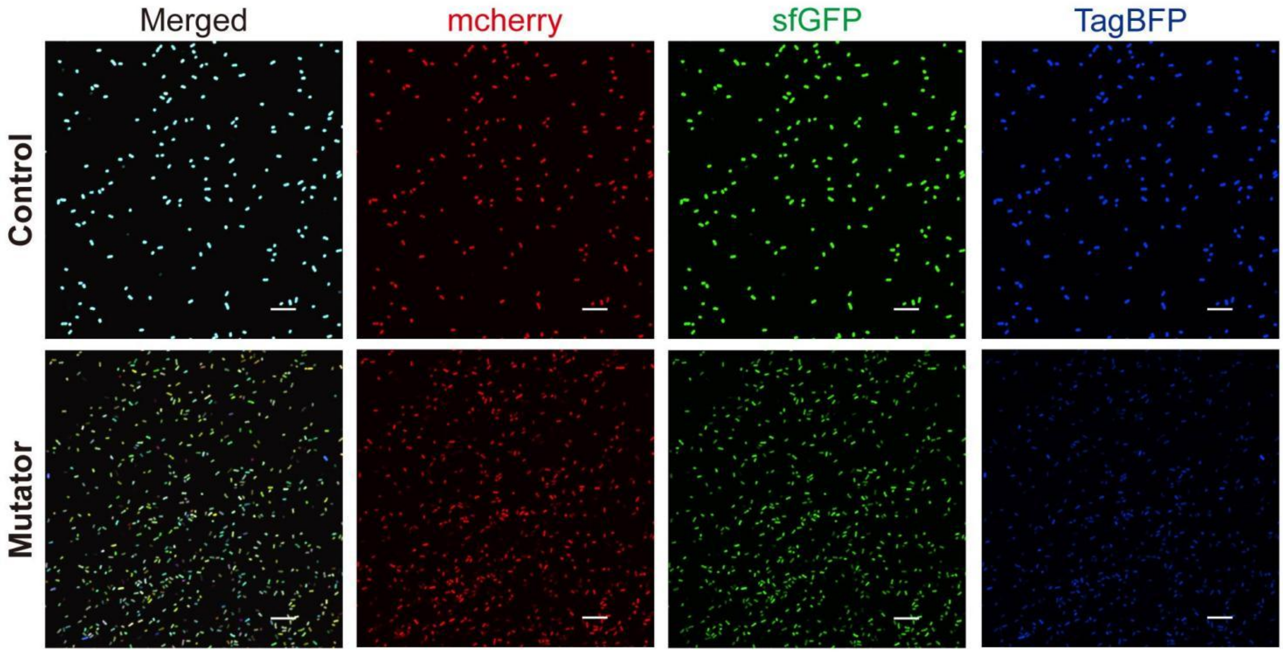

Supplementary Fig. 21 Mutagenesis of fluorescent proteins generates colorful cells of *H. bluephagenesis*.

Confocal microscopy study was performed to observe color changes in *H. bluephagenesis* using the control plasmid pMT0-MmP1 and the mutator plasmid pMT23opt-MmP1 in 50MM medium across the green, red, and blue channels. Scale bar = 10 μm. The experiment was repeated three times with similar results.

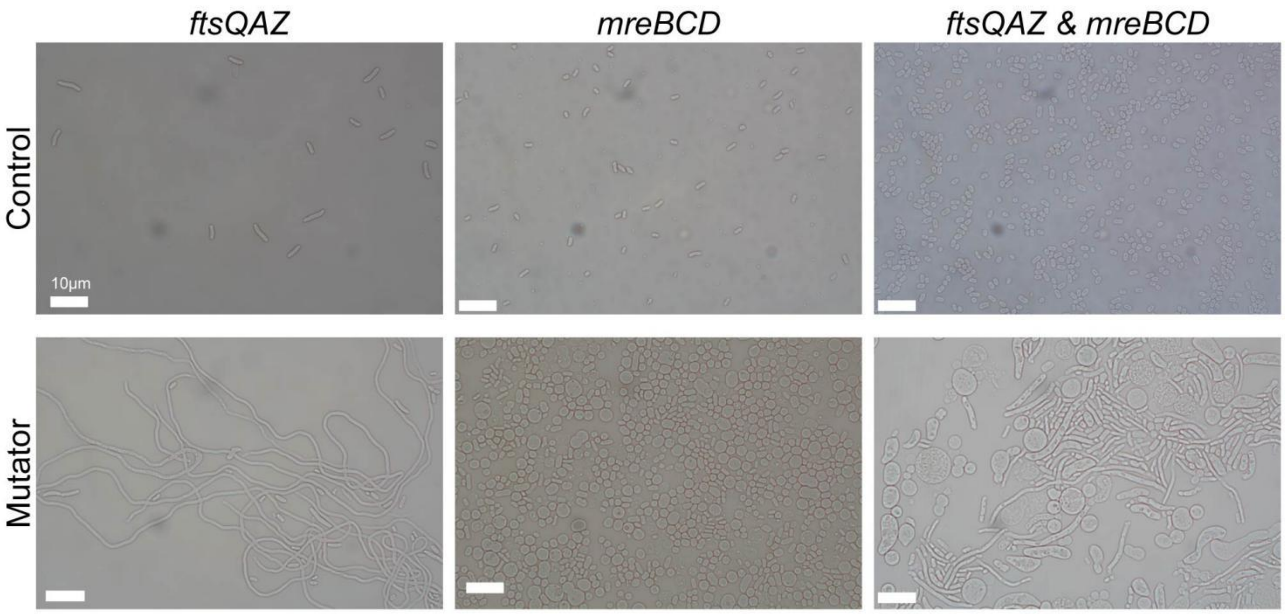

Supplementary Fig. 22 Mutagenesis of cytoskeleton and cell division-related proteins generates shape diversity in *H. bluephagenesis*.

Optical microscopy analysis was conducted to observe the shape changes in three strains in 60LB medium: *H. bluephagenesis* TD01-*mreBCD*-P_{MmP1}, TD01-*ftsQAZ*-P_{MmP1}, and TD01-*mreBCD&ftsQAZ*-P_{MmP1}, utilizing the control plasmid pMT0-MmP1 and the mutator plasmid pMT23opt-MmP1. Scale bar = 10 μm. The experiment was repeated three times with similar results.

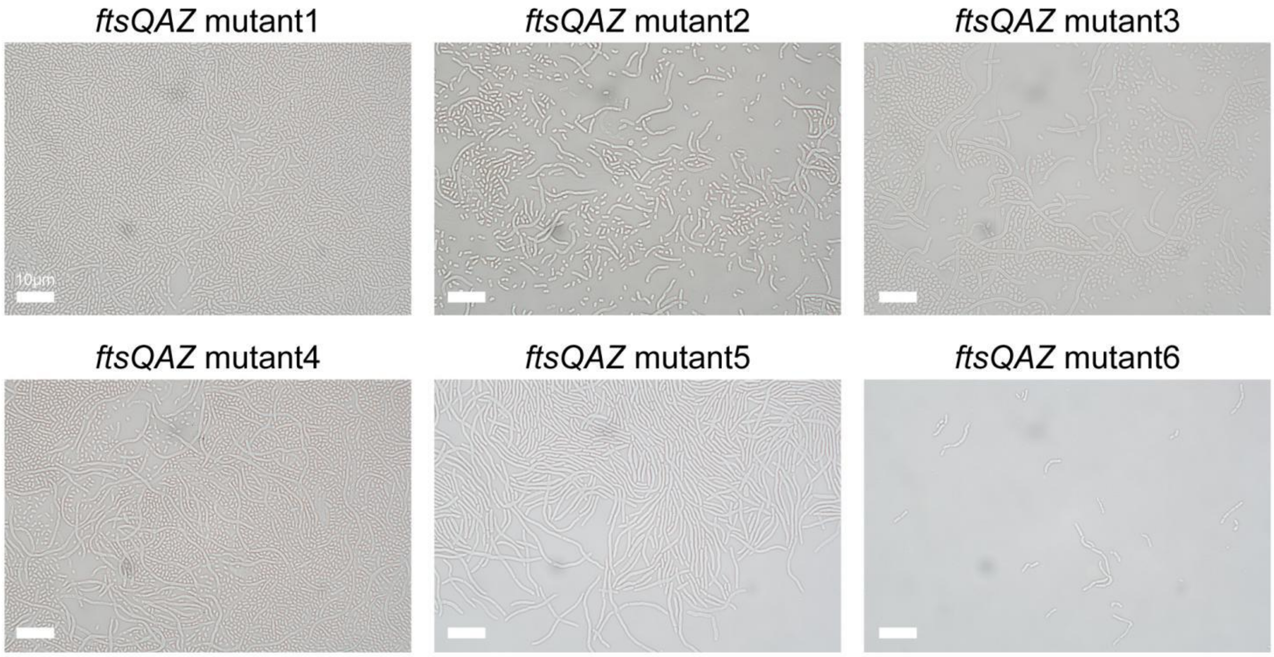

Supplementary Fig. 23 Optical microscopy analysis of *ftsQAZ* mutants from *H. bluephagenesis*.

Six *ftsQAZ* mutants were selected from *H. bluephagenesis* after mutagenesis using the pMT23opt-MmP1 mutator in the 60LB medium. Optical microscopy analysis of *ftsQAZ* mutants revealed elongated rod shapes. Scale bar = 10 μm. Sequencing analysis of six *ftsQAZ* mutants in FtsQ, FtsA, and FtsZ can be found in Supplementary Table 6. The experiment was repeated three times with similar results.

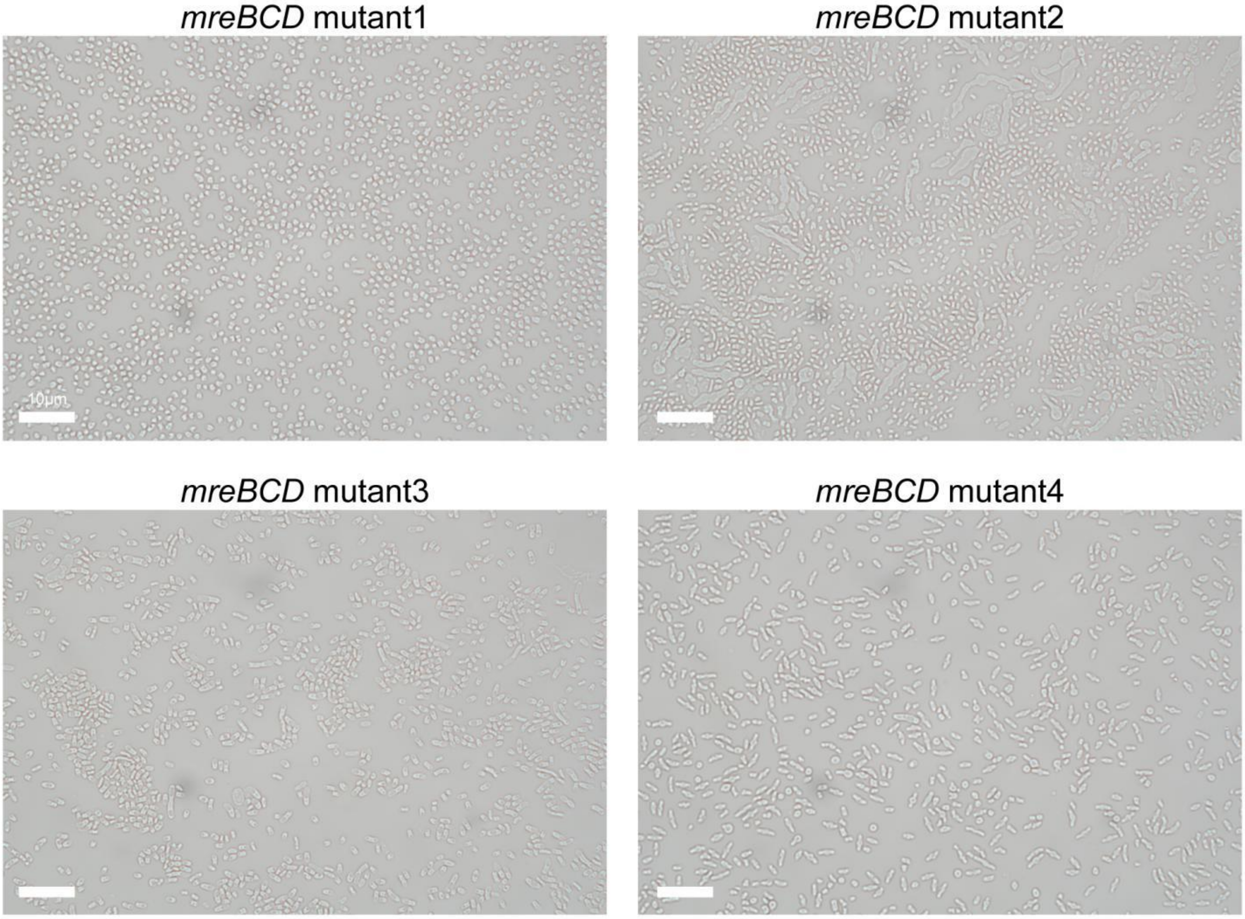

Supplementary Fig. 24 Optical microscopy study of *mreBCD* mutants from *H. bluephagenesis*. Four *mreBCD* mutants were selected from *H. bluephagenesis* after mutagenesis using the pMT23opt-MmP1 mutator in the 60LB medium. Optical microscopy analysis of *mreBCD* mutants revealed spherical shapes. Scale bar = 10 µm. Sequencing analysis of four *mreBCD* mutants in MreB, MreC, and MreD can be found in Supplementary Table 7. The experiment was repeated three times with similar results.

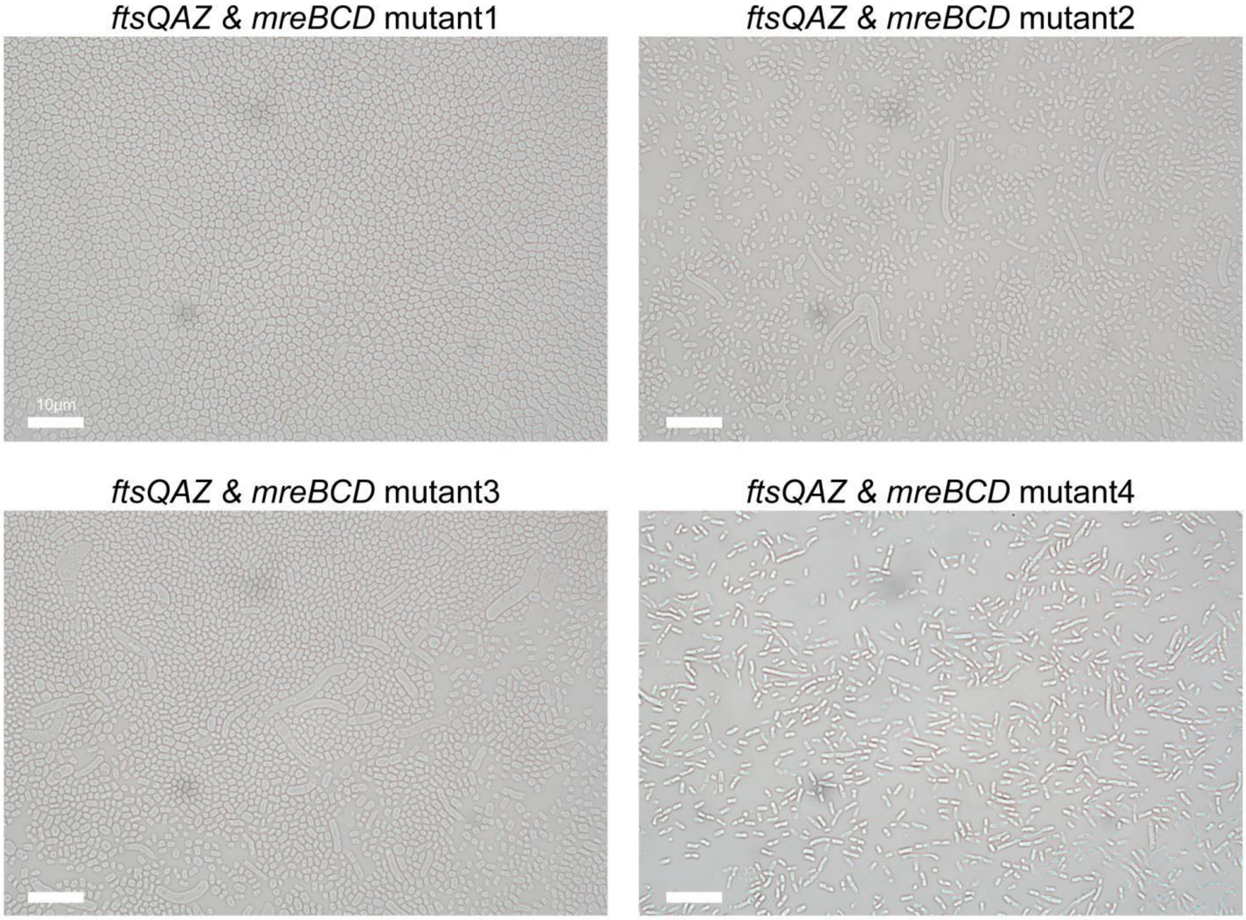

Supplementary Fig. 25 Optical microscopy study of *mreBCD* & *ftsQAZ* mutants from *H. bluephagenesis*.

Four *mreBCD* & *ftsQAZ* mutants were selected from *H. bluephagenesis* after mutagenesis using the pMT23opt-MmP1 mutator in 60LB medium. Optical microscopy analysis of *mreBCD* & *ftsQAZ* mutants revealed spherical, elongated rods and irregular shapes. Scale bar = 10 µm. Sequencing analysis of four *mreBCD & ftsQAZ mutants* in FtsQ, FtsA, FtsZ, MreB, MreC, and MreD can be found in Supplementary Tables 8 and 9. The experiment was repeated three times with similar results.

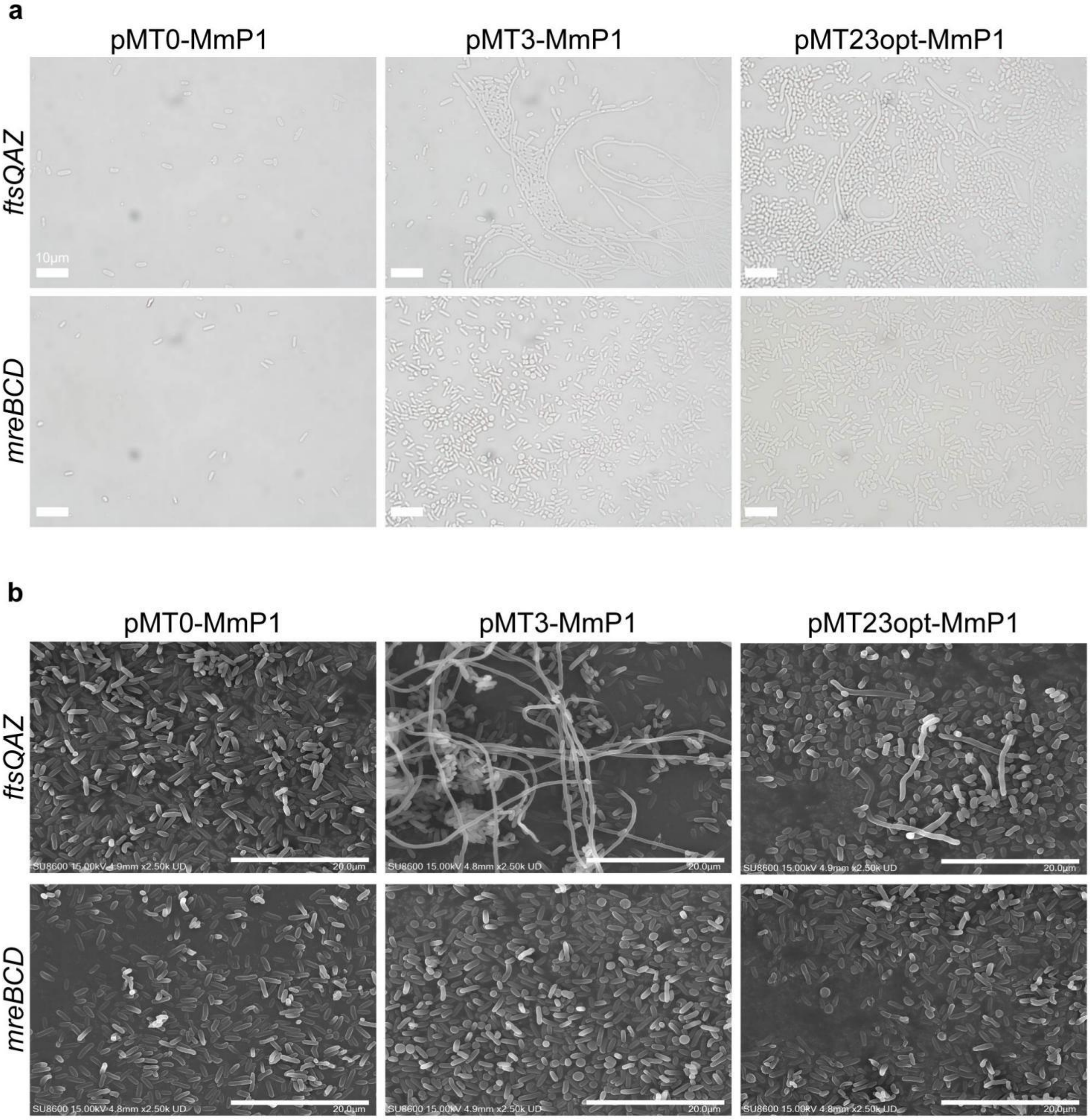

Supplementary Fig. 26 Mutagenesis of cytoskeleton and cell division-related proteins generates shape diversity in *E. coli*.

Optical microscopy (a) and scanning electron microscopy (b) analyses were performed to observe the shape changes in two strains in 10LB medium: *E. coli* MG1655-*mreBCD*-P_{MmP1} and MG1655-*ftsQAZ*-P_{MmP1}, utilizing the control plasmid pMT0-MmP1 and the mutator plasmid pMT3-MmP1 and pMT23opt-MmP1. Scale bars for (a) and (b) are 10 μm and 20 μm, respectively. The experiment was repeated three times with similar results.

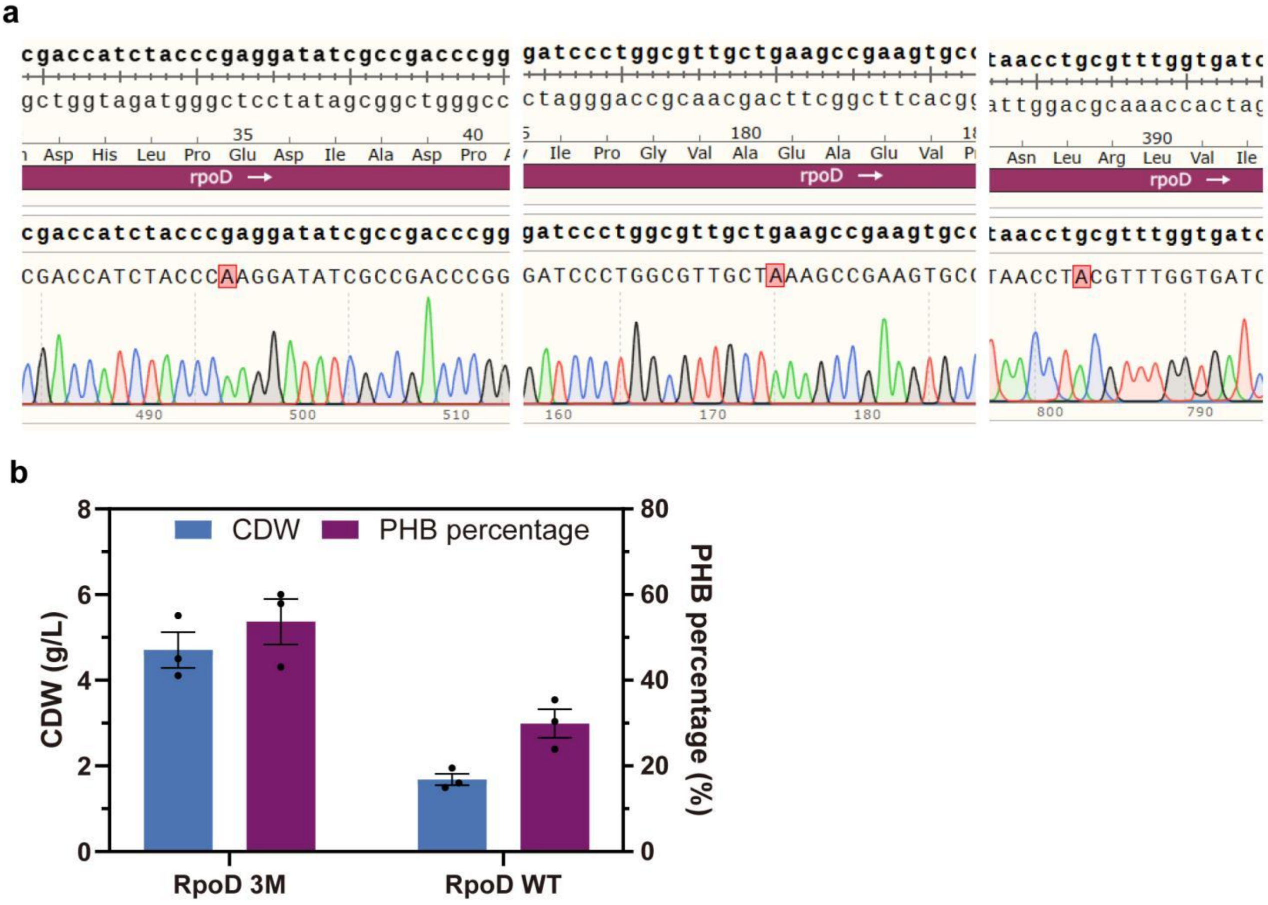

Supplementary Fig. 27 Mutagenesis of sigma 70 factor RpoD for higher L-Arg tolerance in *H. bluephagenesis.*

a Sequencing of RpoD 3M mutant. **b** The shake flask results of RpoD 3M and RpoD WT strains. *n* = 3, which represents three independent replicates of the experiment. CDW represents cell dry weight.

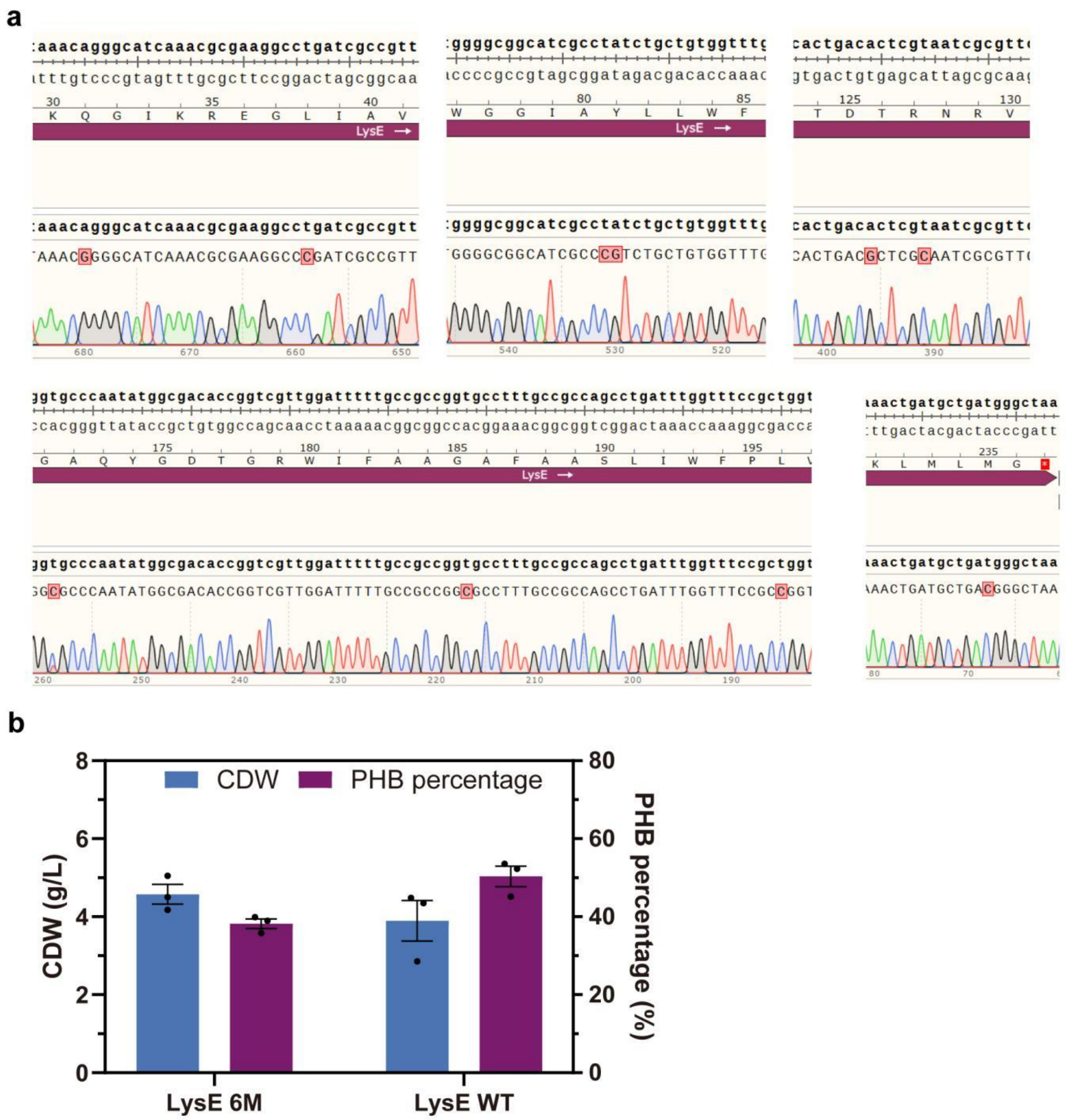

Supplementary Fig. 28 Mutagenesis of LysE exporter for higher L-Arg tolerance in *H. bluephagenesis.*

a Sequencing of LysE 6M mutant. **b** The shake flask results of LysE 6M and LysE WT strains. n = 3, which represents three independent replicates of the experiment.

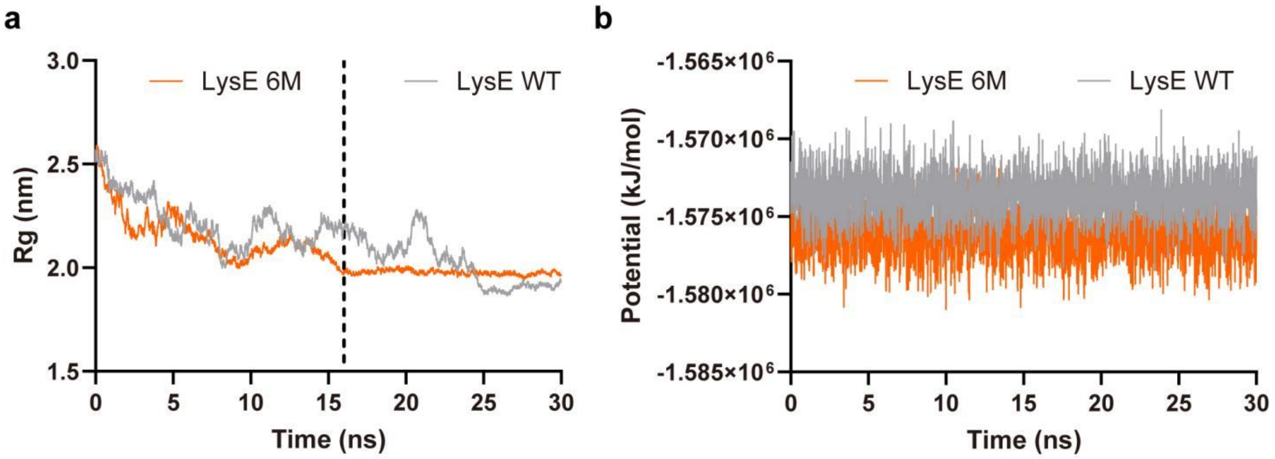

Supplementary Fig. 29 The radius of gyration (a) and potential energy (b) of the LysE 6M mutant and wild-type LysE with L-Arg.

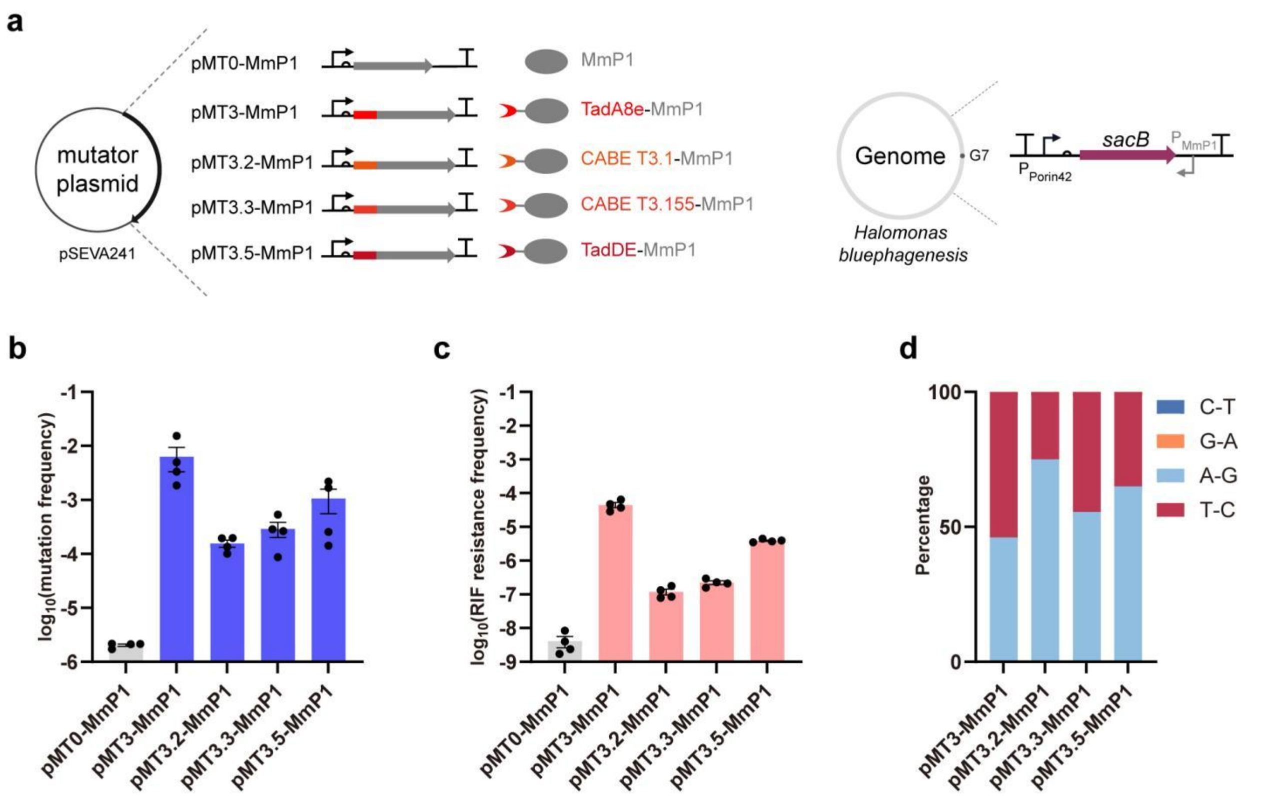

Supplementary Fig. 30 Construction and characterization of dual mutators based on TadA variants and MmP1 RNAP.

a Design of three dual mutators based on the TadA variants CABE T3.1, CABE T3.155, and TadDE. **b** The on-target mutation rate of three dual mutators, as assessed using the *sacB*-based detection method (*n* = 4 independent experiments). **c** The off-target mutation frequency of three dual mutators (*n* = 4 independent experiments). **d** The mutation types in *sacB* after randomly picking up eight colonies from each group for colony PCR and DNA sequencing (*n* = 8 independent experiments). Data are presented as mean values (bars), standard errors (error bars), and individual values (black dots).

Reference

1. Simon, R., Priefer, U. & Pühler, A. A broad host range mobilization system for *in vivo* genetic engineering: transposon mutagenesis in Gram negative bacteria. *Bio. Technol.* **1**, 784-791 (1983).
2. Blattner, F.R. *et al.* The complete genome sequence of *Escherichia coli* K-12. *Science* **277**, 1453-1462 (1997).
3. Tan, D., Xue, Y.-S., Aibaidula, G. & Chen, G.-Q. Unsterile and continuous production of polyhydroxybutyrate by *Halomonas* TD01. *Bioresour. Technol.* **102**, 8130-8136 (2011).
4. Martínez-García, E. *et al.* SEVA 4.0: an update of the Standard European Vector Architecture database for advanced analysis and programming of bacterial phenotypes. *Nucleic Acids Res.* **51**, D1558-D1567 (2023).
5. Ren, K., Zhao, Y., Chen, G.-Q., Ao, X. & Wu, Q. Construction of a stable expression system based on the endogenous hbpB/hbpC toxin–antitoxin system of *Halomonas bluephagenesis*. *ACS Synth. Biol.* **13**, 61-67 (2024).
6. Zhao, H. *et al.* Novel T7-like expression systems used for *Halomonas*. *Metab. Eng.* **39**, 128-140 (2017).
7. Rogozin, I.B. *et al.* Evolution and diversification of lamprey antigen receptors: evidence for involvement of an AID-APOBEC family cytosine deaminase. *Nat. Immunol.* **8**, 647-656 (2007).
8. Thuronyi, B.W. *et al.* Continuous evolution of base editors with expanded target compatibility and improved activity. *Nat. Biotechnol.* **37**, 1070-1079 (2019).
9. Mol, C.D. *et al.* Crystal structure of human uracil-DNA glycosylase in complex with a protein inhibitor: Protein mimicry of DNA. *Cell* **82**, 701-708 (1995).
10. Gaudelli, N.M. *et al.* Programmable base editing of A•T to G•C in genomic DNA without DNA cleavage. *Nature* **551**, 464-471 (2017).
11. Richter, M.F. *et al.* Phage-assisted evolution of an adenine base editor with improved Cas domain compatibility and activity. *Nat. Biotechnol.* **38**, 883-891 (2020).
12. Yan, D. *et al.* High-efficiency and multiplex adenine base editing in plants using new TadA variants. *Mol. Plant* **14**, 722-731 (2021).
13. Neugebauer, M.E. *et al.* Evolution of an adenine base editor into a small, efficient cytosine base editor with low off-target activity. *Nat. Biotechnol.* **41**, 673-685 (2023).
14. Lam, D.K. *et al.* Improved cytosine base editors generated from TadA variants. *Nat. Biotechnol.* **41**, 686-697 (2023).
