# Supplementary material for: An orthogonal transcription mutation system generating all transition mutations for accelerated protein evolution in vivo
Source: Nat Commun. 2025 Jul 1;16:6041. doi: 10.1038/s41467-025-61354-4 (PMC12218169; doi:10.1038/s41467-025-61354-4)
Supplement: Supplementary file 3 — Reporting Summary [file 41467_2025_61354_MOESM3_ESM.pdf]

nature portfolio

Corresponding author(s): Guo-qiang Chen

Last updated by author(s): Jun 7, 2025

Reporting Summary

Nature Portfolio wishes to improve the reproducibility of the work that we publish. This form provides structure for consistency and transparency in reporting. For further information on Nature Portfolio policies, see our Editorial Policies and the Editorial Policy Checklist.

Statistics

For all statistical analyses, confirm that the following items are present in the figure legend, table legend, main text, or Methods section.

n/a	Confirmed	
☐	☒	The exact sample size (*n*) for each experimental group/condition, given as a discrete number and unit of measurement
☐	☒	A statement on whether measurements were taken from distinct samples or whether the same sample was measured repeatedly
☐	☒	The statistical test(s) used AND whether they are one- or two-sided<br>*Only common tests should be described solely by name; describe more complex techniques in the Methods section.*
☒	☐	A description of all covariates tested
☒	☐	A description of any assumptions or corrections, such as tests of normality and adjustment for multiple comparisons
☐	☒	A full description of the statistical parameters including central tendency (e.g. means) or other basic estimates (e.g. regression coefficient) AND variation (e.g. standard deviation) or associated estimates of uncertainty (e.g. confidence intervals)
☐	☒	For null hypothesis testing, the test statistic (e.g. *F*, *t*, *r*) with confidence intervals, effect sizes, degrees of freedom and *P* value noted<br>*Give P values as exact values whenever suitable.*
☒	☐	For Bayesian analysis, information on the choice of priors and Markov chain Monte Carlo settings
☒	☐	For hierarchical and complex designs, identification of the appropriate level for tests and full reporting of outcomes
☒	☐	Estimates of effect sizes (e.g. Cohen's *d*, Pearson's *r*), indicating how they were calculated

Our web collection on statistics for biologists contains articles on many of the points above.

Software and code

Policy information about availability of computer code

Data collection	All data were collected by commercial equipments and softwares shown in Methods and Materials section.
Data analysis	The three-dimensional structures of LysE, RpoD, and their mutants were predicted using AlphaFold2.0. Molecular docking of small molecule ligand (L-Arg) and the receptor protein LysE was performed using AutoDock Vina 1.2.2, and the docking results were visualized using ChimeraX 1.8 software. Molecular dynamics simulations were carried out using GROMACS 2024.1 software. Genetic design and sequence reading were performed by Snap Gene (v6.0.2). Flow cytometer data were analyzed by FlowJo v7.6 software. NIS-Elements Viewer 5.21 was used to process pictures of fluorescence change. All data were analyzed and graphed by GraphPad Prism v8, Adobe lllustrator 2024, and Microsoft 2016 (Excel, Word, PowerPoint).

For manuscripts utilizing custom algorithms or software that are central to the research but not yet described in published literature, software must be made available to editors and reviewers. We strongly encourage code deposition in a community repository (e.g. GitHub). See the Nature Portfolio guidelines for submitting code & software for further information.

Data

Policy information about availability of data

All manuscripts must include a data availability statement. This statement should provide the following information, where applicable:

- Accession codes, unique identifiers, or web links for publicly available datasets
- A description of any restrictions on data availability
- For clinical datasets or third party data, please ensure that the statement adheres to our policy

Data that support the findings of this work can be found in the main manuscript and in the Supplementary information. The source data underlying Figures 2c-h, 3b, 3d-f, 4c-h, 4j-l, 5b, 5d-f, 5h-l, 6, 9 and Supplementary Tables 1-3 and Supplementary Figures 1c, 3, 4c, 6, 8, 9b, 10-16, 27b, 28b, 29a, 29a-b, 30b-d. There is no restriction on the data associated with this study. DNA sequencing data generated in this study have been deposited in the NCBI Sequence Read Archive database under accession code PRJNA1272322. All data related to this study can be found in the main manuscript and the supplementary materials. Source data are provided with this paper.

Research involving human participants, their data, or biological material

Policy information about studies with human participants or human data. See also policy information about sex, gender (identity/presentation), and sexual orientation and race, ethnicity and racism.

Reporting on sex and gender	The experiments did not involve any human participants.
Reporting on race, ethnicity, or other socially relevant groupings	The experiments did not involve any race, ethnicity, or other socially relevant groupings.
Population characteristics	The experiments did not involve population characteristics.
Recruitment	No recruitment was included in this study.
Ethics oversight	No ethics oversight was needed in this study.

Note that full information on the approval of the study protocol must also be provided in the manuscript.

Field-specific reporting

Please select the one below that is the best fit for your research. If you are not sure, read the appropriate sections before making your selection.

☒ Life sciences ☐ Behavioural & social sciences ☐ Ecological, evolutionary & environmental sciences

For a reference copy of the document with all sections, see nature.com/documents/nr-reporting-summary-flat.pdf

Life sciences study design

All studies must disclose on these points even when the disclosure is negative.

Sample size	No statistical methods were used to predetermine sample size. Data from at least three independent experiments (n>=3) was analysed because it is the standard in the field and the minimal size to determine statistical differences.
Data exclusions	No data were excluded from the analysis.
Replication	Data from at least three independent experiments (i.e., independent microbial cultures or strains) was collected for analysis. All attempts at replication were successful.
Randomization	We don't need to allocate samples into experimental groups in essence, because this is not relevant to our study. Individual colonies from each bacterial strain for DNA sequencing were selected randomly.
Blinding	Blinding was not performed in the study. As mutation frequency calculation through colony counting provides objective data independent of observer bias, blinding procedures were not required.

Reporting for specific materials, systems and methods

We require information from authors about some types of materials, experimental systems and methods used in many studies. Here, indicate whether each material, system or method listed is relevant to your study. If you are not sure if a list item applies to your research, read the appropriate section before selecting a response.

Materials & experimental systems

n/a	Involved in the study
☒	☐ Antibodies
☒	☐ Eukaryotic cell lines
☒	☐ Palaeontology and archaeology
☒	☐ Animals and other organisms
☒	☐ Clinical data
☒	☐ Dual use research of concern
☒	☐ Plants

Methods

n/a	Involved in the study
☒	☐ ChIP-seq
☐	☒ Flow cytometry
☒	☐ MRI-based neuroimaging

Plants

Seed stocks: No seed stocks were involved in this study.

Novel plant genotypes: No plant genotypes were found in this study.

Authentication: No authentication

Flow Cytometry

Plots

Confirm that:

☒ The axis labels state the marker and fluorochrome used (e.g. CD4-FITC).

☒ The axis scales are clearly visible. Include numbers along axes only for bottom left plot of group (a 'group' is an analysis of identical markers).

☒ All plots are contour plots with outliers or pseudocolor plots.

☒ A numerical value for number of cells or percentage (with statistics) is provided.

Methodology

Sample preparation: To verify whether the phage RNAP mutators can transcribe the target gene normally, the expression level of sfGFP in H. bluephagenesis strains was analyzed by flow cytometry. H. bluephagenesis strains harboring a target plasmid expressing sfGFP and a mutator plasmid were cultured in the 60LB medium containing Cm and Spe at 37°C for 10 h. Then, the seed cultures were diluted 100-fold in the 50MM medium containing 200 mg/L IPTG inducer. Subsequently, 2 μL of culture solution was diluted 100-fold in 200 μL of PBS for flow cytometry analysis.

Instrument: Flow Cytometer (LSRFortessa4, BD bioscience, USA)

Software: FlowJo (v7.6) software was used to process the raw data for obtaining mean values of fluorescence intensity and percentage of fluorescent positive cells.

Cell population abundance: 2 μL culture was added into 200 μL PBS solution. The diluted culture was recorded by flow cytometer at the rate of 0.5 μL/s for 20 seconds at least 10,000 cell counts were captured.

Gating strategy: FITC (488 nm excitation light), FSC (forward scatter), and SSC (side scatter) channels were recorded. All captured events were used for fluorescence analysis.

☒ Tick this box to confirm that a figure exemplifying the gating strategy is provided in the Supplementary Information.
